# Evaluating two small-sample corrections for fixed-effects standard errors and inferences in multilevel models with heteroscedastic, unbalanced, clustered data

**DOI:** 10.3758/s13428-023-02325-9

**Published:** 2024-02-06

**Authors:** Yichi Zhang, Mark H. C. Lai

**Affiliations:** Department Psychology, University of Southern California, 3620 South McClintock Ave., Los Angeles, CA 90089-1061 USA

**Keywords:** Multilevel modeling, Variance estimation, Heteroscedasticity, Small samples, Unbalanced clusters

## Abstract

Multilevel modeling (MLM) is commonly used in psychological research to model clustered data. However, data in applied research usually violate one of the essential assumptions of MLM—homogeneity of variance. While the fixed-effect estimates produced by the maximum likelihood method remain unbiased, the standard errors for the fixed effects are misestimated, resulting in inaccurate inferences and inflated or deflated type I error rates. To correct the bias in fixed effects standard errors and provide valid inferences, small-sample corrections such as the Kenward-Roger (KR) adjustment and the adjusted cluster-robust standard errors (CR-SEs) with the Satterthwaite approximation for *t* tests have been used. The current study compares KR with random slope (RS) models and the adjusted CR-SEs with ordinary least squares (OLS), random intercept (RI) and RS models to analyze small, heteroscedastic, clustered data using a Monte Carlo simulation. Results show the KR procedure with RS models has large biases and inflated type I error rates for between-cluster effects in the presence of level 2 heteroscedasticity. In contrast, the adjusted CR-SEs generally yield results with acceptable biases and maintain type I error rates close to the nominal level for all examined models. Thus, when the interest is only in within-cluster effect, any model with the adjusted CR-SEs could be used. However, when the interest is to make accurate inferences of the between-cluster effect, researchers should use the adjusted CR-SEs with RS to have higher power and guard against unmodeled heterogeneity. We reanalyzed an example in Snijders & Bosker (2012) to demonstrate the use of the adjusted CR-SEs with different models.

**Supplementary Information:**

The online version contains supplementary material available at 10.3758/s13428-023-02325-9.

Clustered data is common in psychological and educational research. For example, in educational studies, students are nested within schools; in longitudinal studies, observations are nested within individuals. In such data, errors are correlated within clusters but not across clusters. However, statistical models such as linear regression assume errors to be independent and follow identical distributions (i.i.d.). Ignoring the within-cluster error correlation results in underestimating standard errors, misleadingly narrower confidence intervals, larger *t* statistics, and smaller *p* values (Colin & Miller, 2015). A widely used method for analyzing clustered data is multilevel model (MLM; Raudenbush and Bryk, 2002; Snijders and Bosker, 2012), while alternative methods such as cluster-robust standard errors (CR-SEs) with ordinary least square (OLS) estimates, generalized estimating equations (GEE), and Taylor series linearization can also be used to account for correlated errors (McNeish, Stapleton, & Silverman, 2017).

One prominent issue of MLM is small-sample bias (McNeish, 2016). With small samples, the estimates of the fixed effect standard errors tend to be downwardly biased, leading to inflated type I error rates. Two small-sample corrections have been proposed to correct for fixed-effect standard errors: Kenward-Roger (KR) (Kenward & Roger, 1997) and the small-sample adjusted CR-SEs (Pustejovsky & Tipton, 2018). Typical MLM also imposes the homogeneity of variance assumption. For example, a two-level random slopes model requires equal variances of errors across observations and equal variances of random effects across clusters (Snijders & Bosker, 2012). In reality, this assumption is often violated due to data missing at unequal rates across treatment groups (Spilke, Piepho, & Hu, 2005). Though prior research has assessed the performance of adjusted CR-SEs in the context of small-sample data, to our knowledge, our paper is the first to examine the performance of the adjusted CR-SEs on data with heteroscedasticity at two levels and compare it with Kenward–Roger, the default and recommended estimation for most small-sample studies.

Moreover, real data often have unbalanced cluster sizes, which may be paired with unequal variances. The co-occurrence of unbalanced cluster sizes and heteroscedasticity could result in underestimated standard error estimates of fixed effects, and this influence is more problematic with small samples (Raudenbush & Bryk, 2002). These issues bias the variance estimation given by MLM, potentially leading to erroneous conclusions for hypothesis testing (Raudenbush & Bryk, 2002; Snijders & Bosker, 2012). However, the issue of heteroscedasticity with MLM has been overlooked in existing literature, so little is known about ignoring heteroscedasticity on fixed-effects inferences, especially for small unbalanced samples. The current study aims to examine how the common small-sample corrections for standard error estimators would perform on clustered data with heteroscedasticity at two levels.

This manuscript first introduces MLM and its assumptions, as well as the related standard error estimators and hypothesis testing procedures. Next, small-sample bias, the correction for standard error estimation, and associated inferences are reviewed, specifically the KR correction. Then, we review previous literature on how heterogeneous variance and unbalanced cluster sizes influence statistical inferences, as well as the corresponding standard error adjustments for small samples, the adjusted CR-SEs. A Monte Carlo simulation is conducted to compare the two standard error estimators with various models in small samples with heteroscedasticity and unbalanced cluster sizes. We illustrated the use of each method in an applied example that investigates students’ language test performance with IQ scores. Lastly, we provided some recommendations and directions for future studies.

## Multilevel models

### Overview

With clustered data, MLM is a popular method in psychological and educational research because of its advantages in making inferences for cluster level parameters and modeling cross-level interactions (McNeish et al., 2017). MLM accounts for the hierarchical nature of data by differentiating the effects of explanatory variables into fixed effects and random effects. Fixed effects describe the shared relationship between the outcome variable and the explanatory variable across clusters and observations. Random effects represent the cluster-specific deviations in intercepts and slopes from the shared regression line (Snijders & Bosker, 2012).

Consider a two-level MLM with $p - 1$ predictors and $J$ clusters in matrix form:

1 $$\begin{aligned} \varvec{\textbf{Y}}_j = \varvec{\textbf{X}}_j \varvec{\mathbf {\upgamma }}+ \varvec{\textbf{Z}}_j \varvec{\textbf{u}}_j + \varvec{\mathbf {\epsilon }}_j, \end{aligned}$$

where $\varvec{\textbf{Y}}_j$ is a column vector with length $n_j$ (assume $n_j$ people in the $j$th cluster), and $\varvec{\textbf{X}}_j$ is the design matrix with dimension $n_j \times p$ for fixed effects (rows are observations, columns are the fixed effects of the intercept and predictors). In this equation, $\varvec{\textbf{Z}}_j$ is an $n_j \times q$ design matrix for $q$ random effects (rows are observations, columns are the random intercept and random slopes). Let $\varvec{\mathbf {\upgamma }}$ be a $p \times 1$ vector of fixed effects, $\varvec{\textbf{u}}_j$ be a $q \times 1$ vector of random effects, and $\varvec{\mathbf {\epsilon }}_j$ be a $n_j \times 1$ vector of level 1 error terms. The random effects ($\varvec{\textbf{u}}_j$) are assumed to follow a multivariate normal distribution, $\varvec{\textbf{u}}_j \sim N(\varvec{\textbf{0}}, \varvec{\textbf{T}})$, where $\varvec{\textbf{T}}$ is the covariance matrix of random effects. The level 1 error is assumed to follow independent normal distributions, $\varvec{\mathbf {\epsilon }}_{j} \sim N(0, \upsigma ^2 \varvec{\textbf{I}}_{n_j})$.

Similar to linear regression analysis, the assumptions of MLM include linearity, normality, and homoscedasticity (Maas & Hox, 2004; Snijders & Bosker, 2012). In MLM specifically, homoscedasticity means that the variances of errors and random effects should be equal across observations and clusters (Snijders & Bosker, 2012). However, this assumption can be relaxed by adding the relations between predictor variables and variance into the model (Goldstein, 2010; Raudenbush & Bryk, 2002; Snijders & Bosker, 2012).

### Estimation methods

The two major estimation methods in MLM are maximum likelihood (ML) estimation and restricted maximum likelihood (REML) estimation, both of which generate the most probable value of model parameters given the observed data (Raudenbush & Bryk, 2002). In this section, we first review the details of these two estimators and discuss their limitations, then introduce the CR-SEs, a variance estimator that can be used in conjunction with REML estimates.

#### Maximum likelihood estimation and restricted maximum likelihood estimation

ML gives parameter estimates with the highest likelihood by simultaneously estimating variance components and fixed effects components (Raudenbush & Bryk, 2002). Since these two components are dependent, an iterative approach, such as the expectation maximization (EM) algorithm, is often used to find the maximum likelihood estimates. Specifically, the fixed effects are estimated assuming the random effects are missing in the initial iteration, then used for estimating variance components. In the second iteration, the variance estimates from the first iteration are used to update the fixed-effect estimates. This process continues until the estimates stay the same (McNeish, 2017). However, this procedure ignores the variability of fixed-effect estimates and does not account for the loss of degrees of freedom in fixed-effects estimation. These problems are more pronounced with small samples because sampling variability is large and impacts of degrees of freedom are substantial. Thus, although the parameter estimates are still unbiased, the variance components are underestimated, resulting in underestimated standard errors and biased inference (Browne & Draper, 2006).

REML improves the performance of the ML method by separating the estimation of fixed effects and variance components (McNeish, 2017). Specifically, REML first transforms the data to eliminate the fixed effects and then estimates the variance components. Next, the generalized least square (GLS) method is used to estimate the fixed effects. The variance of $\varvec{\textbf{Y}}_j$ in Eq. 1 can be written as (Raudenbush and Bryk, 2002, p. 278)

$$\begin{aligned} \text {Var}(\varvec{\textbf{Y}}_j) = \text {Var}(\varvec{\textbf{Z}}_j \varvec{\textbf{u}}_j + \varvec{\mathbf {\epsilon }}_j) = \varvec{\textbf{V}}_j =\varvec{\textbf{Z}}_j \varvec{\textbf{T}} \varvec{\textbf{Z}}_j^\top +\upsigma ^2\varvec{\textbf{I}}_{n_j}. \end{aligned}$$

GLS assumes variance components are known, so the regression coefficients can be written as

$$\begin{aligned} {\varvec{\hat{\mathbf {\upgamma }}}} = (\sum _{j=1}^{J}\varvec{\textbf{X}}_j^\top \varvec{\textbf{V}}_j^{-1} \varvec{\textbf{X}}_j)^{-1}\sum _{j=1}^{J} \varvec{\textbf{X}}_j^\top \varvec{\textbf{V}}_j^{-1} \varvec{\textbf{Y}}_j, \end{aligned}$$

where $J$ is the number of clusters. The variance of regression coefficients is

2 $$\begin{aligned} \text {Var}^{GLS}(\varvec{\hat{\mathbf {\upgamma }}}) = (\sum _{j=1}^{J} \varvec{\textbf{X}}_j^\top \varvec{\textbf{V}}_j^{-1} \varvec{\textbf{X}}_j)^{-1}. \end{aligned}$$

Note that both ML and REML assume the random effects and errors have equal variances (i.e., homoscedasticity). When this assumption is violated, the parameter estimates are still asymptotically unbiased, but the standard error estimates are inaccurate (Raudenbush & Bryk, 2002; Snijders & Bosker, 2012).

### Statistical inferences

In addition to parameter estimates, hypothesis testing is also of interest. In MLM, the single parameter test statistic $\frac{\hat{\upgamma }}{\text {SE}(\hat{\upgamma })}$ under the null hypothesis $H_0:\upgamma =0$ follows approximately a $t$ distribution (Raudenbush & Bryk, 2002; Snijders & Bosker, 2012), with the degrees of freedom (*df*) equal to the total number of units at the specified level minus the number of predictors at the same level minus one. When the *df* is large, the $t$ distribution is close to a normal distribution.

Multiparameter test for MLM includes the multivariate Wald test and the likelihood-ratio test (Snijders & Bosker, 2012). The multivariate Wald test is only applicable to test fixed-effects parameters, whereas the likelihood-ratio test can be used in both multiparameter test and test of random-effects parameters by examining whether the difference in deviance from the models is statistically significant. Further details of hypothesis testing in MLM can be found at Snijders and Bosker (2012).

### Problems with small samples and unequal variances

In psychological and educational research, small samples with unequal variances are common. Below we discuss the small-sample estimation bias and proposed corrections in the existing literature. Heteroscedasticity and unbalanced cluster sizes are also discussed in detail, with a focus on their influences on standard error estimates and finite sample inferences.

### Small-sample size

It is common to have small samples for clustered data collected in psychological research. A widely used criterion for small samples is 30 clusters with a size of 30 observations (Kreft & Leeuw, 1998). According to a review by McNeish (2016), using the above criterion, 20% of multilevel models and 30% cluster randomized trials have small samples. Dedrick et al. (2009) reviewed 99 previous social science studies using multilevel models and found that 20% of them have sample sizes smaller than the suggested cut-offs.

In multilevel models, a small sample size is more complex than single-level linear regression. Since the upper-level sample size is always smaller than the lower-level sample size (Maas & Hox, 2005), the upper-level sample size determines whether the data suffers small-sample bias (Snijders, 1993). When the number of clusters is small, there is little information about between-group variations, so it is difficult to estimate the variance parameters (Gelman & Hill, 2006).

Ignoring the small-sample bias results in biased estimates for parameters and inaccurate inference. Maas and Hox (2005) found that when the number of clusters is smaller than 100, the standard errors for the second-level variance components can be underestimated. Other studies generally found that the fixed effects standard errors were also downwardly biased when the number of clusters is below 25 (McNeish & Stapleton, 2016).

### Small-sample corrections for covariance matrix estimation and inferences

To improve the performance of standard error estimators in small samples, some corrections are proposed to further adjust the standard errors and the degrees of freedom for the hypothesis tests, such as the KR correction.

#### Kenward-Roger correction

The KR correction can be used for making inferences of fixed-effect parameters with small samples estimated with REML. The first step of KR is to ensure that the test statistic derived from REML is accurate. Although REML improves the variance estimates in small samples compared to ML, the standard error estimates can still be biased. Kackar and Harville (1984) used a Taylor Series expansion to correct for this bias. Moreover, REML ignores the variability of variance components when fitting the GLS model to get fixed-effects estimates. Thus, KR incorporated Kackar and Harville’s approximation and performed a second Taylor series expansion to account for the variability in computing the *t* test statistic (Kenward & Roger, 1997; McNeish, 2017). The second step of KR is to correct the degrees of freedom of the *t* test using a method based on the Satterthwaite approximation (Kenward & Roger, 1997). By adjusting both the test statistic and the degrees of freedom, KR keeps the type I error rate at a nominal level in small, homoscedastic samples. However, it is unknown how KR would perform with the existence of heteroscedasticity.

### Heteroscedasticity

We now turn to homogeneity of variance, which is another essential assumption for MLM. Unequal variances of errors across observations or unequal variance of random effects across clusters is called heteroscedasticity (Snijders & Bosker, 2012). When the effects of predictors vary across clusters but are treated as fixed, then the unmodeled random effects are shown as heteroscedastic error variance (Snijders & Bosker, 2012). Some causes for heteroscedasticity are omitted predictor variables, omitted random effects, coding errors in multiple clusters, and nonnormal data with heavy tails (Raudenbush & Bryk, 2002). The current study focuses on the omitted relations between predictors and variance at two levels.

To better illustrate the cluster-level heteroscedasticity, here we provided a hypothetical example. Imagine a researcher is interested in the effectiveness of a stress management intervention for college students. Students are nested within classrooms and are randomly assigned to two groups: treatment and control. The research questions are whether the stress reductions vary across treatment and control conditions (level 2 predictor), and whether students with higher self-esteem (group mean-centered level 1 predictor) have higher stress reduction. Homoscedasticity of random intercepts means the classroom means of stress reduction for students who have the average self-esteem have the same variability regardless of the group (treatment/control) that these classrooms are assigned to. In contrast, heteroscedasticity of random intercepts means the classroom means of stress reduction for students who have the average self-esteem have a larger or smaller variability for treatment groups than that of the control group. For random slopes, homoscedasticity holds when the variability of the relationships between self-esteem and stress reductions are similar for classrooms that are assigned to treatment group and the control group. If the relationships between self-esteem and stress reductions varies a lot in classrooms that are assigned to treatment group, but do not differ much for classrooms assigned to control group, then it is an example of heteroscedasticity of random slopes.

The influence of heteroscedasticity on parameter estimation and hypothesis tests depends on whether the variance is related to the explanatory variables. If the variance varies randomly, then the influence on coefficients’ point estimates and standard errors is negligible (Raudenbush & Bryk, 2002). However, if homoscedasticity is assumed, but variance depends on the explanatory variable either at level 1 or level 2, then the influence of heteroscedasticity can be serious (Kasim & Raudenbush, 1998; Snijders & Bosker, 2012). In addition, when heteroscedasticity is present at both level 1 and level 2 (Guillermo Vallejo, Fernández, Cuesta, & Livacic-Rojas, 2015), the fixed-effects estimates are unbiased, but the associated standard errors are biased. The direction of bias depends on the pairing of heteroscedastic variance and cluster sizes.

#### Standard error adjustments

Some adjustments have been proposed to correct the standard error estimates given by the ML or REML when distributional assumptions, such as homoscedasticity, are violated. One adjustment is to apply the robust standard errors, which are generated by first estimating the regression model with OLS and then adjusting the standard errors using the Satterthwaite estimation proposed by White (1980). The resulting standard errors are called robust standard errors or heteroscedastic-robust standard errors. Zeger, Liang, and Albert (1988) applied this estimator to clustered data, which generates the CR-SEs. CR-SEs do not place assumptions on the correlation structure of errors; instead they assume the observations can be grouped into mutually independent clusters (Pustejovsky & Tipton, 2018). Although MLM already accounts for the error correlation by specifying the random components, CR-SEs can still be applied to MLM to account for unmodeled heterogeneity (Hox et al., 2010; Huang & Li, 2021; McNeish et al., 2017; Raudenbush & Bryk, 2002; Yuan & Bentler, 2002). Raudenbush and Bryk (2002) extended the heteroscedastic-robust variance estimator on GLS estimates (p. 278),

3 $$\begin{aligned} \text {Var}^{Robust}(\varvec{\hat{\mathbf {\upgamma }}})= &  \text {Var}^{GLS}(\varvec{\hat{\mathbf {\upgamma }}}) \\ &  \sum _{j=1}^{J} \varvec{\textbf{X}}_j^\top \varvec{\textbf{V}}_j^{-1} (\varvec{\textbf{Y}}_j \!- \varvec{\textbf{X}}_j \varvec{\hat{\mathbf {\upgamma }}})(\varvec{\textbf{Y}}_j \!- \varvec{\textbf{X}}_j \varvec{\hat{\mathbf {\upgamma }}})^\top \varvec{\textbf{X}}_j \varvec{\textbf{V}}_j^{-1}\text {Var}^{GLS}(\varvec{\hat{\mathbf {\upgamma }}}) \nonumber \end{aligned}$$

The details of this heteroscedastic-robust estimator for MLM can be found at Raudenbush and Bryk (2002). In short, if assumptions of homoscedasticity are violated, GLS will produce unbiased regression coefficients and biased standard error estimates. However, $\text {Var}^{GLS}$ given by Eq. 2 estimates the variability in the regression coefficients with bias, whereas $\text {Var}^{Robust}$ uses a correction matrix based on observed residuals to generate more accurate standard error estimates. If residuals follow homoscedasticity assumptions, both $\text {Var}^{GLS}$ and $\text {Var}^{Robust}$ are consistent variance estimators but $\text {Var}^{GLS}$ will be more efficient. With the presence of heteroscedasticity, $\text {Var}^{Robust}$ has been found to be more accurate (Huang & Li, 2021; Raudenbush & Bryk, 2002).

#### Adjusted cluster-robust standard errors

The CR-SEs described in Eq. 3 are based on the original HC0 estimator derived by White (1980), which may underestimate the covariance matrix when the sample size is small (MacKinnon, 2012). MacKinnon and White (1985) proposed several modifications such as HC1, HC2, and HC3, which are also called the heteroscedasticity consistent covariance matrix estimator (HCCME). These modifications have been generalized to clustered data labeled as CR1, CR2, and CR3 (Bell & Mccaffrey, 2002; Colin & Miller, 2015; Pustejovsky & Tipton, 2018). Specifically, assuming there are $J$ clusters in the data set, CR1 multiplies the estimated variance matrix with $\sqrt{\frac{J}{J-1}}$ (Colin & Miller, 2015). However, this adjustment still leads to underestimated variance when the cluster sizes are unbalanced. Bell and Mccaffrey (2002) proposed the CR2 or bias-reduced linearization (BRL) estimator, an extension of the HC2 variance estimator. The BRL estimator assumes the residuals follow a “working model” specified by researchers, and it defines adjustment matrices for the variance estimator to yield unbiased estimates (Pustejovsky & Tipton, 2018). With cluster-randomized trials, the working model could be that observations within the same clusters have some shared relationships, but observations from different clusters are assumed to be independent (Huang & Li, 2021). Mancl and DeRouen (2001) proposed CR3, which leads to the delete-one-cluster jackknife estimate of the variance. However, CR3 can over-correct the standard error estimates, resulting in over-rejections for small samples (Pustejovsky & Tipton, 2018), so the literature tends to suggest using CR2 (Colin & Miller, 2015).

CR-SEs are developed to be robust to clustering and heteroscedasticity, and the BRL estimator largely improves its performance in small samples. However, the *df* that determines the critical values is also an important factor for accurate small-sample inferences (Bell & Mccaffrey, 2002; Pustejovsky & Tipton, 2018). Bell and Mccaffrey (2002) extended the Satterthwaite *df* approximation for *t* tests that compare sample means with heteroscedastic variances to clustered data ($\text {df}_\text {BRL}$). Previous studies have shown using the BRL adjusted CR-SEs with $\text {df}_\text {BRL}$ performed well for small, clustered data set with heteroscedastic variances (Bell & Mccaffrey, 2002; Huang & Li, 2021; Satterthwaite, 1946). Imbens and Kolesár (2016) argued the BRL estimator and the $\text {df}_\text {BRL}$ should always be used even for moderate sample sizes.

However, BRL is undefined in some models and the magnitude of BRL adjustments also depends on the specific estimator that is used. Thus, Pustejovsky and Tipton (2018) extended the BRL estimator to commonly used models, which is referred to as the adjusted CR-SEs in this paper, and implemented it to R package *clubSandwich* (Pustejovsky, 2023). Pustejovsky and Tipton (2018) found the adjusted CR-SEs with estimated degrees of freedom, ($\text {df}_\text {ABRL}$), performed well in small data sets with unbalanced cluster sizes. The type I error rates were controlled close to the nominal level across all conditions. Huang and Li (2021) investigated the performance of the adjusted CR-SEs and the $\text {df}_\text {ABRL}$) (which they called CR2 and $\text {dof}_\text {BM}$) with the OLS regression on data with few clusters. They found that the adjusted CR-SEs with $\text {df}_\text {ABRL}$ generated acceptable standard errors and coverage probabilities. Using the adjusted CR-SEs with the OLS regression also has the advantage of not requiring the specification of random effects and the assumption that the model is correctly specified. Moreover, Pustejovsky and Tipton (2018) used single-level models with CR-SEs, so it is unclear how the adjusted CR-SEs will perform in conjunction with REML estimates from MLM with heteroscedasticity and unbalanced cluster sizes. To our knowledge, the performance of the adjusted CR-SEs with OLS regression and MLM for small, heteroscedastic data has not been compared. Thus, one goal of the current study is to investigate whether both methods would yield acceptable estimates and accurate inferences for data with few clusters.

Note that the CR-SEs or the adjusted CR-SEs are only robust to clustering and heteroscedasticity, meaning that a small departure from normality or the presence of outliers can seriously influence its performance. This issue is revisited in the Discussion.

#### Coexistence of unbalanced data and heteroscedasiticity

One understudied topic in MLM is unbalanced cluster size, which can result from certain experimental designs, such as cross-over designs or unequal numbers of missing data (Spilke et al., 2005). Heteroscedasticity can pair with unbalanced sample sizes because the imbalance might be due to preexisting group differences, generating differences in both group means and group variances (Blanca, Alarcón, Arnau, Bono, & Bendayan, 2018). There are abundant studies in the literature of analysis of variance (ANOVA) discussing this relationship (Blanca et al., 2018; Vallejo et al., 2010).

The coexistence of heteroscedasticity and unbalanced cluster sizes in MLM has a more serious impact when the sample size is small (Raudenbush & Bryk, 2002). Korendijk, Maas, Moerbeek, and Van der Heijden (2008) showed that in the presence of the second-level heteroscedasticity and unbalanced cluster sizes, the standard errors for the second-level fixed and variance components were underestimated when the level 2 sample size was small. Kasim and Raudenbush (1998) found that when the number of clusters is small, the standard errors are sensitive to heteroscedasticity.

Despite much research on the small-sample bias for MLM and some on the influence of heteroscedasticity on MLM estimations, little is known about how the small-sample standard error estimators of MLM would perform with heteroscedastic variances. Thus, one goal of the current study is to compare KR and the adjusted CR-SES with $\text {df}_\text {ABRL}$ when using with MLM on data that have heteroscedasticity paired with unbalanced cluster sizes. Note the adjusted CR-SEs and $\text {df}_\text {ABRL}$ can be used with both the random intercepts model (RI) and the random slopes model (RS). Although KR is not designed to accommodate heteroscedasticity, we included KR as a comparison method for conditions when only small sample, but not heteroscedasticity, is accounted for. By comparing these two small-sample corrections for standard errors, we could figure out whether correctly specifying the model structure would have more benefits.

### Current study

To sum up, estimation and inferences of fixed-effect coefficients are usually of interest in applied research. However, social and behavioral research often involves small samples with heteroscedastic variances and unbalanced cluster sizes, which can bias the standard errors for fixed-effects estimates. The current study uses Monte Carlo simulation to compare the performance of two small-sample corrections for standard error estimators, KR and the adjusted CR-SEs, in inferences of fixed-effect coefficients for small, unbalanced samples with heteroscedastic variances at two levels. In addition, we examine whether adjusted CR-SEs give different results when combining with either OLS, RI, and RS models.

One major contribution of the current study is we extensively studied the performance of small-sample corrections for standard error estimators under heteroscedasticity. Although past studies, such as Huang and Li (2021), compared different types of CR-SEs with variants of *df* and various models, they did not investigate the performance of adjusted CR-SEs used with MLM in the existence of heteroscedasticity at two levels. Another unique contribution is that we compared four options (RS and KR, RS and adjusted CR-SEs with $\text {df}_\text {ABRL}$, RI and adjusted CR-SEs with $\text {df}_\text {ABRL}$, OLS and adjusted CR-SEs with $\text {df}_\text {ABRL}$) for analyzing small clustered data and examined the influence of missing random slopes on type I error rates and power.^1^ Comparing the above-mentioned four options could help researchers select the best robust and small-sample method while incorporating their knowledge of the data structure.

## Methods

A two-level random slope model with one predictor at level 1 ($x_{ij}$) and one predictor at level 2 ($z_j$),

4 $$\begin{aligned} y_{ij}= &  \upgamma _{00} + \upgamma _{01} z_j + \upgamma _{10}x_{ij} + \upgamma _{11} z_j x_{ij} + {\tilde{\uplambda }}_j (z_j) u_{0j}\nonumber \\ &  + {\tilde{\uplambda }}_j (z_j) u_{1j}x_{ij} + {\tilde{\uplambda }}_j (x_{ij})e_{ij}, \end{aligned}$$

was used to generate the simulated data sets. Here, $x_{ij}$ does not have between-cluster variance so the intraclass correlation ICC$_{X} = 0$, and ${\tilde{\uplambda }}_j$ is a function for heteroscedasticity that will be described later. The data was analyzed using a regular two-level random slope model,

5 $$\begin{aligned} y_{ij} = \upgamma _{00} + \upgamma _{01} z_j + \upgamma _{10}x_{ij}+ \upgamma _{11} z_jx_{ij} + u_{0j} + u_{1j} x_{ij} + e_{ij}. \end{aligned}$$

We set the parameter values based on the simulation conducted by Maas and Hox (2005). Specifically, the grand intercept $\upgamma _{00}$ was set to 1. The slopes for within-cluster $\upgamma _{10}$ and for between-cluster $\upgamma _{01}$ were set to 0 when examining type I error rates and 0.3 when examining power. In their paper, Maas and Hox mentioned that 0.3 corresponds to a medium effect size for regression coefficients (Cohen, 1988), so we set all regression coefficients (including cross-level interaction $\upgamma _{11}$) to be 0.3. The error terms $u_{0j}$, $u_{1j}$ and $e_{ij}$ were generated from independent normal distributions with mean 0 and variances $\uptau _0^2$, $\uptau _1^2$ and $\upsigma ^2$ accordingly. $\upsigma ^2$ was set as 1 and $\uptau _{0}^2$ was set based on the conditional intraclass coefficient (ICC) and $\upsigma ^2$. If $\text {ICC} = 0.1$, then $\uptau _{0}^2 = 0.11$, and if $\text {ICC} = 0.3$, then $\uptau _{0}^2 = 0.43$. Following Maas and Hox (2005), $\uptau _0^2$ and $\uptau _1^2$ were set to be equal because a previous study found that the effects for the intercept variance and slope variance on parameter estimates and associated standard error estimates are similar (Busing, 1993). We found consistent results in a small-scale simulation with $\uptau _0^2 > \uptau _1^2$ and provided details of this simulation in Supplemental Materials. We assumed the covariance between the random slope $u_{0j}$ and the random intercept $u_{1j}$ was 0. The predictors $x_{ij}$ and $z_j$ were generated from the standard normal distribution.

Eight conditions were varied in the simulation: (a) number of clusters; (b) average cluster size; (c) ICC; (d) $\upgamma _{10} = \{0, 0.3\}$; (e) $\upgamma _{01} = \{0, 0.3\}$; (f) balanced versus unbalanced clusters; (g) variance patterns (VP) at level 1; and (h) VP at level 2. There were $3 \times 2 \times 2 \times 2 \times 2 \times 2 \times 3 \times 3 = 864$ conditions. For each condition, 2500 data sets were generated, and the simulation was structured using the R package *SimDesign* (Chalmers & Adkins, 2020). We fit the OLS regression using the R function lm, and the RI and RS models using the R package *lme4* (Bates, Mächler, Bolker, & Walker, 2015). The adjusted CR-SEs was applied after fitting the OLS regression (OLS-CRSEs), the RI model (RI-CRSEs), and the RS model (RS-CRSEs) using the R package *clubSandwich* (Pustejovsky, 2023). The KR correction was applied with the RS model (RS-KR) using the R package *lmerTest* (Kuznetsova et al., 2017).

### Design conditions

#### Number of clusters

The simulation included three conditions for the number of clusters ($J$): 15, 30, and 60. We chose 30 and 50 because they are the common number of clusters included in organizational and educational research (Kreft & Leeuw, 1998). We also included conditions with $J = 15$ to evaluate the small-sample performance of the two standard error estimators (Korendijk et al., 2008).

#### Average cluster size

We included average cluster sizes of 5 and 30, as a cluster size of 5 is normal in longitudinal research, and a cluster size of 30 is found to be sufficient for MLM (Maas & Hox, 2005).

#### Intraclass correlation (ICC)

The conditional ICC represents the average correlation between observations in the same cluster (Snijders & Bosker, 2012), which was set as 0.1 and 0.3 in the current study, based on the typical range reported in Hedges and Hedberg (2007). Past simulation studies showed that ICC could significantly influence the accuracy of the estimates and standard errors (Hox & Maas, 2001).

#### Imbalance of cluster size

The cluster sizes were simulated to be balanced (i.e., constant) or unbalanced. For the balanced design, each cluster had the average cluster size. For the unbalanced design, the current study followed Lai (2021), dividing the number of clusters into five strata, so each had $J/5$ clusters. The five strata had sizes of $n/3$, $2n/3$, $n$, $4n/3$, and $5n/3$. The largest clusters were five times the size of the smallest clusters.

#### Variance patterns

The current simulation study examined three variance patterns at levels one and two: VP1 (i.e., equal residual variance across observations within clusters, and equal random effects variance between clusters), VP2 (i.e., the conditional variance of $Y$ given predictor $X$ and $Z$ is largest when the predictors are at the average values), and VP3 (i.e., the conditional variance of $Y$ given predictor $X$ and $Z$ is smallest when the predictors are at the average values) (Wilcox, 2006). The first variance pattern, VP1, represents homoscedasticity, and the latter two variance patterns (VP2, VP3) represent heteroscedasticity. Homoscedasticity served as a baseline condition, where $\uplambda (z_j)$ and $\uplambda (x_{ij})$ in Eq. 4 were set to be 1. Heteroscedasticity data were simulated as functions of cluster sizes $n$ since the unbalanced cluster sizes often influence how estimators react to heteroscedastic data (Blanca et al., 2018; Guillermo Vallejo et al., 2015). Specifically, random effects were simulated using

$$\begin{aligned} {\tilde{\uplambda }}_j (z_j) u_{0j} + {\tilde{\uplambda }}_j (z_j) u_{1j}x_{ij} + {\tilde{\uplambda }}_j (x_{ij})e_{ij}, \end{aligned}$$

where ${\tilde{\uplambda }}_j (z_j) = k_j\sqrt{n_j}\times \uplambda (z_j)$, and ${\tilde{\uplambda }}_j (x_{ij}) = k_j\sqrt{n_j}\times \uplambda (x_{ij})$. Here, $k_j$ is the correction factor that ensures the variance of random effects is the same across conditions, $n_j$ is the number of observations for the $j$th cluster. The first type of heteroscedastic condition VP2 was simulated with $\uplambda (z_j)=|z_j|+1$ and $\uplambda (x_{ij})=|x_{ij}|+1$ such that the variance of $y_{ij}$ is the smallest when predictors are close to their mean. The second type of heteroscedastic condition VP3 was simulated with $\uplambda (z_j)=\frac{1}{|z_j|+1}$ and $\uplambda (x_{ij})=\frac{1}{|x_{ij}|+1}$ such that the variance of $y_{ij}$ is the smallest when predictors are far from their mean. In the added term $k\sqrt{n_j}$, the variance is positively related to the cluster-specific cluster size and the ratio of the variance for the largest cluster to the smallest cluster was $5:1$. This ratio represents a severe violation of the homoscedasticity assumption (Guillermo Vallejo et al., 2015). We further multiplied $\sqrt{n_j}$ by $k_j$ to ensure the average variance of the homoscedasticity condition and the heteroscedasticity condition are approximately equal. When $\bar{n} = 5$, $k_j = 0.25$ with VP2 and $k_j = 0.10$ with VP3. When $\bar{n} = 30$, $k_j = 0.73$ with VP2 and $k_j = 0.30$ with VP3.

### Evaluation criteria

Relative bias, empirical type I error rates, and simulation-based power was used to evaluate the four standard error estimators. Since the previous study found that the point estimates of fixed-effect parameters given by OLS, RI and RS are similar (McNeish et al., 2017), we focused on standard error estimates in this simulation. Specifically, the relative bias for fixed effects standard errors was calculated by $\frac{1}{R}\sum _{r = 1}^{R}\frac{(\hat{\uptheta }_{r}-\uptheta )}{\uptheta }$, where $r = 1, 2,..,R$ represents the number of replications. Since the interest is in comparing standard error estimators, $\hat{\uptheta }$ is the estimated standard error for fixed effects, and $\uptheta$ is the population standard error, calculated as the standard deviation of the fixed-effects parameter estimates across replications. Previous studies have suggested that the relative bias within $\pm 10\%$ is considered negligible (Muthén & Muthén, 2002).

In this study, we focus on single-parameter tests, so the fixed-effect parameters were tested using *t* tests with a significance level of 0.05. The *df* for KR is a scaled version of Satterthwaite correction ($\text {df}_{\text {KR}}$ ; McNeish, 2017), and the df for the adjusted CR-SEs is $\text {df}_{\text {ABRL}}$. The empirical type I error rate was calculated using the proportion of replications that reject the null hypothesis given the null hypothesis is true ($\upgamma _{10}$ or $\upgamma _{01} = 0$). We used Bradley’s liberal criterion to evaluate the performance of standard error estimators on type I error rate. Specifically, if the type I error rate falls between 0.025 and 0.075, it is considered robust. Otherwise, the test is too liberal (above 0.075) or too conservative (below 0.025) (Bradley, 1978).

The simulation-based power was calculated as the proportion of replications that reject the null hypothesis given that the null hypothesis is false ($\upgamma _{10}$ or $\upgamma _{01} = 0.3$). Because the power difference could result from the differences in type I error rates, we also calculated the corrected power proposed by Barr, Levy, Scheepers, and Tily (2013). Specifically, if the type I error rate was inflated, then the 5% quantile of *p* values from the simulation conditions under the null hypothesis was used as a cutoff to calculate power instead of 0.05. In contrast, if the type I error rates were deflated, then 0.05 was still used as the cutoff because adjusting for power would artificially make the test more powerful.

**Table 1:** Relative bias for standard errors of intercept

		ICC = 0.1		ICC = 0.3	
J	Estimator	Balanced	Unbalanced	Balanced	Unbalanced
15	Kenward-Roger RS	0.001	-0.003	−0.009	−0.020
15	Adjusted CR-SEs RI	−0.026	−0.042	−0.026	−0.035
15	Adjusted CR-SEs RS	−0.025	−0.048	−0.021	−0.039
15	Adjusted CR-SEs OLS	−0.028	−0.054	−0.028	−0.060
30	Kenward-Roger RS	−0.006	0.001	−0.012	−0.005
30	Adjusted CR-SEs RI	−0.013	−0.013	−0.016	−0.012
30	Adjusted CR-SEs RS	−0.015	−0.017	−0.015	−0.013
30	Adjusted CR-SEs OLS	−0.014	−0.017	−0.017	−0.021
50	Kenward-Roger RS	−0.001	−0.002	0.003	0.001
50	Adjusted CR-SEs RI	−0.002	−0.004	0.005	0.002
50	Adjusted CR-SEs RS	−0.004	−0.009	0.002	−0.002
50	Adjusted CR-SEs OLS	−0.002	−0.006	0.005	−0.001

*Note.* *J* is the number of clusters; *ICC* represents the intraclass coefficient. *Adjusted CR-SEs* is the abbreviation of the adjusted cluster-robust standard errors. *OLS* represents the ordinary least squares models; *RI* represents the random intercept models; *RS* represents the random slope models

**Fig. 1:**
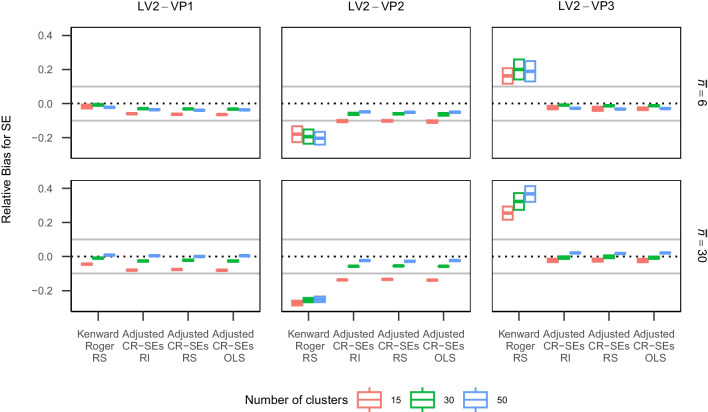
Relative bias for standard errors of between-cluster coefficient ($\upgamma _{01}$) with heteroscedasticity at level 2. Note $\bar{n}$ is the average cluster size, SE means the standard error of the between-cluster coefficient. The *gray lines* represent the lower and upper bounds of acceptable values of relative bias for standard errors. *LV2* is the abbreviation of level 2. *VP1* represents homoscedasticity; *VP2* represents when the conditional variance of outcome variable is largest when the predictors are at the average values; *VP3* represents when the conditional variance of outcome variable is smallest when the predictors are at the average values. *Adjusted CR-SEs* is the abbreviation of the adjusted cluster-robust standard errors. *OLS* represents the ordinary least squares models; *RI* represents the random intercept models; *RS* represents the random slope models

## Results

Results showed the influences of small and unbalanced cluster sizes on fixed-effect parameters were small and consistent for all conditions. Table 1 depicts the relative bias for standard errors of intercept that illustrates this pattern. Since the average cluster size, $\bar{n}$, had a negligible influence on estimating standard errors of the between-cluster effect, we presented the overall results without differentiating the values of $\bar{n}$. As expected, the examined methods performed slightly better when the cluster sizes were balanced compared to unbalanced.

The impact of heteroscedasticity depends on the specific fixed-effect parameters and the level that heteroscedasticity exists. Below we present the simulation results relevant to heteroscedasticity.

### Relative bias for standard errors

The standard errors for the within-cluster coefficient ($\upgamma _{10}$) were estimated with bias less than $10\%$ for most conditions regardless of corrections used and heteroscedasticity (see Fig. S1 in Supplemental Materials). In contrast, when estimating the between-cluster coefficient ($\upgamma _{01}$), level 2 heteroscedasticity had appreciable influences (see Fig. 1 and Table 2). In this condition, the KR corrected standard errors were underestimated in VP2 conditions, and overestimated in VP3 conditions, with bias ranging from -0.30 to 0.42 (*M* = 0.00, *SD* = 0.20). The adjusted CR-SEs were underestimated for most conditions with a magnitude of bias from -0.19 to 0.03 (*M* = -0.05, *SD* = 0.05) for OLS regression, from -0.16 to 0.03 (*M* = -0.05, *SD* = 0.04) for RI, and from -0.17 to 0.02 (*M* = -0.05, *SD* = 0.04) for RS. With level 1 heteroscedasticity, all examined methods slightly underestimated the standard error of $\upgamma _{01}$ with bias less than $10\%$ (see Fig. S2 in Supplemental Materials).

**Table 2:** Relative bias for standard errors of between-cluster effect

			Level 2 VP1		Level 2 VP2		Level 2 VP3	
ICC	J	Estimator	Balanced	Unbalanced	Balanced	Unbalanced	Balanced	Unbalanced
0.3	15	Kenward-Roger RS	−0.035	−0.047	$\mathbf {-0.253}$	**-0.268**	**0.246**	**0.216**
0.3	15	Adjusted CR-SEs RI	−0.068	−0.085	$\mathbf {-0.124}$	$\mathbf {-0.153}$	−0.014	−0.037
0.3	15	Adjusted CR-SEs RS	−0.067	−0.090	$\mathbf {-0.121}$	$\mathbf {-0.157}$	−0.016	−0.045
0.3	15	Adjusted CR-SEs OLS	−0.071	$\mathbf {-0.108}$	$\mathbf {-0.126}$	$\mathbf {-0.182}$	−0.018	−0.061
0.3	30	Kenward-Roger RS	−0.009	−0.001	$\mathbf {-0.249}$	$\mathbf {-0.241}$	**0.310**	**0.318**
0.3	30	Adjusted CR-SEs RI	−0.027	−0.017	−0.061	−0.060	−0.003	0.010
0.3	30	Adjusted CR-SEs RS	−0.024	−0.022	−0.059	−0.063	0.000	0.003
0.3	30	Adjusted CR-SEs OLS	−0.028	−0.030	−0.063	−0.083	−0.004	−0.007
0.3	50	Kenward-Roger RS	−0.009	−0.011	$\mathbf {-0.252}$	$\mathbf {-0.255}$	**0.325**	**0.325**
0.3	50	Adjusted CR-SEs RI	−0.017	−0.023	−0.039	−0.053	−0.003	−0.006
0.3	50	Adjusted CR-SEs RS	−0.021	−0.025	−0.042	−0.054	−0.008	−0.012
0.3	50	Adjusted CR-SEs OLS	−0.018	−0.031	−0.040	−0.068	−0.004	−0.012

*Note.* *J* is the number of clusters; *ICC* represents the intraclass coefficient. *Adjusted CR-SEs* is the abbreviation of the adjusted cluster-robust standard errors. *OLS* represents the ordinary least squares models; *RI* represents the random intercept models; *RS* represents the random slope models. *VP1* represents homoscedasticity; *VP2* represents when the conditional variance of outcome variable is largest when the predictors are at the average values; *VP3* represents when the conditional variance of outcome variable is smallest when the predictors are at the average values. *Bolded numbers* suggest the relative bias is larger than 10%

**Table 3:** Type I error rates and simulation-based power for detecting between-cluster effect

			Level 2 VP1			Level 2 VP2			Level 2 VP3		
ICC	J	Estimator	Type I	Power	Power’	Type I	Power	Power’	Type I	Power	Power’
0.3	15	Kenward-Roger RS	0.050	0.279	0.275	0.129	0.312	0.181	0.016	0.219	0.219
0.3	15	Adjusted CR-SEs RI	0.041	0.214	0.214	0.057	0.173	0.157	0.026	0.257	0.257
0.3	15	Adjusted CR-SEs RS	0.044	0.223	0.222	0.057	0.178	0.163	0.026	0.269	0.269
0.3	15	Adjusted CR-SEs OLS	0.044	0.208	0.208	0.060	0.168	0.148	0.026	0.232	0.232
0.3	30	Kenward-Roger RS	0.048	0.541	0.541	0.126	0.513	0.331	0.012	0.473	0.473
0.3	30	Adjusted CR-SEs RI	0.045	0.475	0.475	0.054	0.321	0.309	0.032	0.607	0.607
0.3	30	Adjusted CR-SEs RS	0.045	0.489	0.489	0.054	0.330	0.319	0.034	0.626	0.626
0.3	30	Adjusted CR-SEs OLS	0.046	0.452	0.452	0.056	0.297	0.281	0.033	0.550	0.550
0.3	50	Kenward-Roger RS	0.050	0.761	0.758	0.134	0.677	0.494	0.010	0.729	0.729
0.3	50	Adjusted CR-SEs RI	0.050	0.713	0.707	0.058	0.474	0.445	0.038	0.830	0.830
0.3	50	Adjusted CR-SEs RS	0.051	0.736	0.732	0.060	0.494	0.463	0.039	0.846	0.846
0.3	50	Adjusted CR-SEs OLS	0.050	0.682	0.674	0.061	0.433	0.400	0.039	0.780	0.780

*Note.* *Type I* represents type I error rates and *power’* represents corrected power. *J* is the number of clusters; *ICC* represents the intraclass coefficient. *Adjusted CR-SEs* is the abbreviation of the adjusted cluster-robust standard errors. *OLS* represents the ordinary least squares models; *RI* represents the random intercept models; *RS* represents the random slope models. *VP1* represents homoscedasticity; *VP2* represents when the conditional variance of outcome variable is largest when the predictors are at the average values; *VP3* represents when the conditional variance of outcome variable is smallest when the predictors are at the average values

**Table 4:** Type I error rates and simulation-based power for detecting within-cluster effect

			Level 2 VP1			Level 2 VP2			Level 2 VP3		
ICC	J	Estimator	Type I	Power	Power’	Type I	Power	Power’	Type I	Power	Power’
0.3	15	Kenward-Roger RS	0.043	0.276	0.276	0.043	0.277	0.277	0.046	0.250	0.249
0.3	15	Adjusted CR-SEs RI	0.053	0.258	0.246	0.052	0.260	0.253	0.050	0.214	0.209
0.3	15	Adjusted CR-SEs RS	0.048	0.278	0.274	0.049	0.287	0.281	0.046	0.246	0.242
0.3	15	Adjusted CR-SEs OLS	0.052	0.252	0.242	0.051	0.254	0.246	0.050	0.211	0.207
0.3	30	Kenward-Roger RS	0.053	0.533	0.523	0.053	0.507	0.495	0.053	0.476	0.465
0.3	30	Adjusted CR-SEs RI	0.057	0.485	0.462	0.058	0.451	0.426	0.056	0.410	0.389
0.3	30	Adjusted CR-SEs RS	0.054	0.535	0.522	0.055	0.514	0.496	0.053	0.477	0.464
0.3	30	Adjusted CR-SEs OLS	0.055	0.472	0.458	0.056	0.441	0.423	0.053	0.395	0.382
0.3	50	Kenward-Roger RS	0.047	0.745	0.744	0.049	0.711	0.708	0.048	0.669	0.665
0.3	50	Adjusted CR-SEs RI	0.051	0.682	0.676	0.052	0.629	0.621	0.050	0.570	0.561
0.3	50	Adjusted CR-SEs RS	0.047	0.746	0.743	0.051	0.713	0.706	0.047	0.669	0.665
0.3	50	Adjusted CR-SEs OLS	0.050	0.673	0.668	0.051	0.624	0.621	0.049	0.560	0.557

*Note.* *Type I* represents type I error rates and *power’* represents corrected power. *J* is the number of clusters; *ICC* represents the intraclass coefficient. *Adjusted CR-SEs* is the abbreviation of the adjusted cluster-robust standard errors. *OLS* represents the ordinary least squares models; *RI* represents the random intercept models; *RS* represents the random slope models. *VP1* represents homoscedasticity; *VP2* represents when the conditional variance of outcome variable is largest when the predictors are at the average values; *VP3* represents when the conditional variance of outcome variable is smallest when the predictors are at the average values

### Type I error rate

Both KR and the adjusted CR-SEs controlled the type I error rates close to the nominal level ($\upalpha = 0.05$) with heteroscedasticity at level 1 (see Table S3 in Supplemental Materials). With level 2 heteroscedasticity, for the between-cluster effect ($\upgamma _{01}$), RS-KR had inflated type I error rates with VP2 (ranging from $0.06$ to $0.15$), and deflated type I error rates with VP3 (ranging from $0$ to $0.03$). In contrast, the adjusted CR-SEs controlled the type I error rate well using Bradley’s liberal criterion (see Table 3). For the within-cluster effect ($\upgamma _{10}$) reported in Table 4, both RS-KR and adjusted CR-SEs with examined models performed well and the associated type I error rates were close to the nominal level.

### Power

The simulation-based power was calculated as the proportion of iterations that rejected the null hypothesis when the between-cluster effect or within-cluster effect was 0 (see Table 3 for $\upgamma _{01}$ and Table 4 for $\upgamma _{10}$). The corrected power was the same as the simulation-based power except when power was artificially inflated (VP2). Because patterns were similar in conditions with ICC $= 0.1$ and ICC $= 0.3$, we only presented the results with ICC $= 0.3$ and provided the results with ICC $=0.1$ in the Supplemental Materials (see Tables S1 and S2). Level 1 heteroscedasticity had a negligible impact on power with both KR and the adjusted CR-SEs, whereas level 2 heteroscedasticity had a stronger impact on power with the adjusted CR-SEs. For between-cluster and within-cluster effects ($\upgamma _{01}$, $\upgamma _{10}$) with level 2 heteroscedasticity, RS-KR and RS-CRSEs had highest and comparable power, followed by RI-CRSEs and OLS-CRSEs. The same pattern was shown on the within-cluster effect with level 1 heteroscedasticity. The differences in power across level 2 variance patterns were more significant on the adjusted CR-SEs compared to RS-KR. As shown in Table 3, the adjusted CR-SEs with examined models had lower simulation-based and corrected power than RS-KR with level 2 VP2, but higher power with level 2 VP3.

### Summary

Since the simulation results showed that the adjusted CR-SEs could control the type I error rates close to the nominal level, we suggest using the adjusted CR-SEs rather than KR when estimating the between-cluster effect with the existence of level 2 heteroscedasticity. Due to the near equivalence of standard error estimates as well as type I error rates provided by RS-CRSEs, RI-CRSEs, and OLS-CRSEs, researchers can use OLS-CRSEs to control for the error dependence within clusters without making rigid assumptions inherent in MLMs. If the interest is only in within-cluster coefficients, any of the examined methods could be used. However, if researchers are also interested in between-cluster coefficient estimates, or if the sample is relatively small and the goal is to have higher power, we suggest using the RS-CRSEs as it has the highest power among all examined models.

## Illustrative example

We reanalyzed an example discussed in Snijders and Bosker (2012) to demonstrate the use of the adjusted CR-SEs with OLS, RI, RS models, and RS with KR. The data set came from a study investigating whether school differences exist in students’ language proficiency and arithmetic performance and to what extent school-related factors explain such differences (Brandsma & Knuver, 1989). Data were collected from a random sample of grade 8 students from 250 elementary schools in the Netherlands. To compare the methods in small samples, we further randomly sampled 15 schools with complete observations and focused on language proficiency scores ($M = 41.74$, $SD = 8.87$, range from 16 to 57). The final data set contained 316 observations (female = $48.42\%$, minority = $0.95\%$) from 15 schools with class sizes ranging from 10 to 32.^2^ In addition, socioeconomic status (SES) and verbal IQ were centered in the original data, so their mean was close to 0 in the original data set. In this random sample, students have higher SES than the mean ($M_{SES} = 1.57$, $SD_{SES} = 11.73$) and average IQ at around the mean level ($M_{IQ} = -0.21$, $SD_{IQ} = 2.07$).

**Fig. 2:**
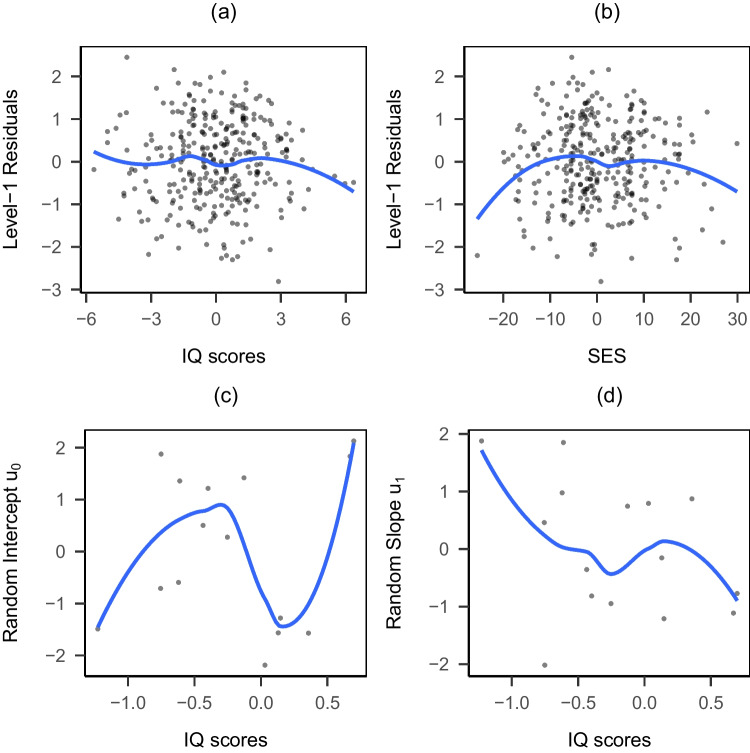
Residual plots for the empirical example. **a** Level 1 residuals across IQ scores. **b** Level 1 residuals across SES. **c** Random intercept residuals across school average IQ scores. **d** Random slope residuals across school average IQ scores

Following Snijders and Bosker (2012), we examined how students’ verbal IQ score, SES, sex, and related class variables predicted their language performance scores. An empty MLM was fit to obtain the unconditional intraclass coefficient, indicating the average correlation of students’ language performance scores from the same school is 0.10. Three models in conjunction with two standard error estimators were compared: OLS with the adjusted CR-SEs, RI with the adjusted CR-SEs, RS with the adjusted CR-SEs and KR.

The statistical models for OLS, RI, and RS, with the language score of the $i$th person in the $j$th cluster as the outcome, are provided below.

**OLS**:

$$\begin{aligned} \text {language}_{ij}= &  \upbeta _0 + \upbeta _1 \overline{\text {IQ}}_{j} + \upbeta _2 (\text {IQ}_{ij} - \overline{\text {IQ}}_{j}) \nonumber \\ &  + \upbeta _3 (\text {SES}_{ij} - \overline{\text {SES}}_{j})+ \upbeta _4 \overline{\text {SES}}_{j} + \epsilon _{i}. \end{aligned}$$

**RI**:

$$\begin{aligned} \text {language}_{ij}= &  \upgamma _{00} \!+ \upgamma _{10} (\text {IQ}_{ij} \!- \overline{\text {IQ}}_{j}) \!+ \upgamma _{11} (\text {SES}_{ij} - \overline{\text {SES}}_{j}) \\ &  + \upgamma _{01}\overline{\text {IQ}}_{j} + \upgamma _{02} \overline{\text {SES}}_{j} + u_{0j} + \epsilon _{ij}. \end{aligned}$$

**RS**:

$$\begin{aligned} \text {language}_{ij}= &  \upgamma _{00} + \upgamma _{10} (\text {IQ}_{ij} \!- \overline{\text {IQ}}_{j}) + \upgamma _{11} (\text {SES}_{ij} \!- \overline{\text {SES}}_{j})\\ &  + \upgamma _{01}\overline{\text {IQ}}_{j} + \upgamma _{02} \overline{\text {SES}}_{j} + u_{0j}\\ &  + u_{1j}(\text {IQ}_{ij} - \overline{\text {IQ}}_{j}) + \epsilon _{ij}. \end{aligned}$$

The adjusted CR-SEs for the OLS regression, RI, and RS were obtained using the coef_test() function from the package *clubSandwich* (Pustejovsky, 2023). KR with the RS was obtained using the summary() function from the package *lmerTest* (Bates et al., 2015). R code for the analysis is provided in Supplemental Materials.

### Checking homoscedasticity

The assumptions for equal variances across observations (level 1 homoscedasticity) and random effects across clusters (level 2 homoscedasticity) were examined using residual plots and the Breusch-Pagan (BP) test (Huang et al., 2022). Figure 2(a) and (b) show the variability of level 1 RS residuals across IQ scores and SES, respectively. The BP test suggests a violation of the homoscedasticity assumption for level 1 IQ scores (*p* = 0.020) but not for level 1 SES scores (*p* = 0.099). Figure 2(c) and (d) show that the variability in random intercepts and random slopes across schools for the school average IQ scores are largest when the school has the IQ close to the overall mean IQ scores, which correspond to VP2 in the simulation. Similarly, the BP test result supports the existence of level 2 heteroscedasticity (*p* = 0.022). Thus, the homoscedasticity assumptions for IQ scores at both levels are violated in this example.

### Comparison of results

Table 5 shows the results of different analytic approaches. All models provided similar coefficients estimates. Specifically, the results showed students IQ ($\upbeta _{\scriptscriptstyle CR-SEs}^{\scriptscriptstyle OLS}$ = 2.189, $\upbeta _{\scriptscriptstyle CR-SEs}^{\scriptscriptstyle RI}$ = 2.189, $\upbeta _{\scriptscriptstyle CR-SEs}^{\scriptscriptstyle RS}$ = 2.174), SES ($\upbeta _{\scriptscriptstyle CR-SEs}^{\scriptscriptstyle OLS}$ = 0.227, $\upbeta _{\scriptscriptstyle CR-SEs}^{\scriptscriptstyle RI}$ = 0.227, $\upbeta _{\scriptscriptstyle CR-SEs}^{\scriptscriptstyle RS}$ = 0.231), school average IQ ($\upbeta _{\scriptscriptstyle CR-SEs}^{\scriptscriptstyle OLS}$ = 3.315, $\upbeta _{\scriptscriptstyle CR-SEs}^{\scriptscriptstyle RI}$ = 2.988, $\upbeta _{\scriptscriptstyle CR-SEs}^{\scriptscriptstyle RS}$ = 2.959), and school average SES ($\upbeta _{\scriptscriptstyle CR-SEs}^{\scriptscriptstyle OLS}$ = 0.104, $\upbeta _{\scriptscriptstyle CR-SEs}^{\scriptscriptstyle RI}$ = 0.122, $\upbeta _{\scriptscriptstyle CR-SEs}^{\scriptscriptstyle RS}$ = 0.063) were positively related to their language performance.

Given the presence of unequal random intercepts variance across schools, the KR corrected SE for school average IQ scores ($SE_{\scriptscriptstyle KR}^{\scriptscriptstyle RS}$ = 1.678) were larger than the adjusted CR-SEs ($SE_{\scriptscriptstyle CR-SEs}^{\scriptscriptstyle RS}$ = 1.539). However, the associated $p$ values corrected by KR ($p_{\scriptscriptstyle KR}^{\scriptscriptstyle RS}$ = 0.104) were lower than by the adjusted CR-SEs ($p_{\scriptscriptstyle CR-SEs}^{\scriptscriptstyle RS}$ = 0.108) due to the difference in df. This finding is consistent with the simulation results, as KR corrected SEs had higher rejection rates for between-cluster effect with level 2 VP2, whereas the adjusted CR-SEs controlled the type I error rates well but tended to underestimate SE. As residuals are homoscedastic across levels of school average SES, the KR corrected SE ($SE_{\scriptscriptstyle KR}^{\scriptscriptstyle RS}$ = 0.128) were larger than the adjusted CR-SEs ($SE_{\scriptscriptstyle CR-SEs}^{\scriptscriptstyle RS}$ = 0.089), and the associated *p* value corrected by KR ($p_{\scriptscriptstyle KR}^{\scriptscriptstyle RS}$ = 0.634) were higher than by CR-SEs ($p_{\scriptscriptstyle CR-SEs}^{\scriptscriptstyle RS}$ = 0.514). This finding was as expected since the simulation showed KR and the adjusted CR-SEs provided comparable rejection rates for predictors with homoscedastic variances. In general, the standard error estimates adjusted by the two estimators in conjunction with the examined models were comparable and the inferences were consistent at the 0.05 significance level.

## Discussion

The simulation results showed the Kenward-Roger adjustment with random slope models (RS-KR) and the adjusted cluster-robust standard errors (CR-SEs) with examined models were unbiased for small, unbalanced samples with homoscedastic variance across observations and random effects across clusters. With level 1 heteroscedasticity (i.e., unequal variances across observations), regardless of examined models, both standard error estimators showed acceptable bias for fixed effect standard error estimates, and controlled the type I error rates close to the stated significance level for between-cluster effects and within-cluster effects.

However, with level 2 heteroscedasticity (i.e., unequal variances for random intercepts and random slopes across clusters), the results depend on the parameters of interest and the standard error adjustments. Our simulation results suggested that the adjusted CR-SEs controlled the type I error rate close to the stated significance level, regardless of examined models and parameters. In contrast, RS-KR controlled the type I error rates well only with within-cluster effect but performed poorly with between-cluster effect. Specifically, the type I error rates were inflated when the conditional variance is largest for predictors at average values (VP2), and deflated when the conditional variance is the smallest when predictors are at average values (VP3).

**Table 5:** Regression coefficients, standard errors, and *p* values from all models

	Adjusted CR-SEs OLS				Adjusted CR-SEs RI				Kenward-Roger RS				Adjusted CR-SEs RS			
	Coef	SE	*df*	*P* value	Coef	SE	*df*	*P* value	Coef	SE	*df*	*P* value	Coef	SE	*df*	*P* value
Intercept	42.262	0.835	6.490	0.000	41.938	0.919	6.857	0.000	42.108	0.839	12.062	0.000	42.108	0.958	7.141	0.000
IQ	2.189	0.320	11.969	0.000	2.189	0.320	11.969	0.000	2.174	0.274	15.076	0.000	2.174	0.313	13.424	0.000
SES	0.227	0.051	10.872	0.001	0.227	0.051	10.872	0.001	0.231	0.040	291.577	0.000	0.231	0.052	10.835	0.001
Mean IQ	3.315	1.639	5.239	0.097	2.988	1.750	5.473	0.143	2.959	1.678	11.875	0.104	2.959	1.539	5.447	0.108
Mean SES	0.104	0.096	5.850	0.321	0.122	0.093	5.067	0.243	0.063	0.128	12.029	0.634	0.063	0.089	5.065	0.514

*Note.* *Adjusted CR-SEs* is the abbreviation of the adjusted cluster-robust standard errors. *OLS* represents the ordinary least squares models; *RI* represents the random intercept models; *RS* represents the random slope models. *Coef* is the coefficient estimate; *SE* is the standard error; *df* is degrees of freedom

The simulation-based power indicated that the RS-KR and adjusted CR-SEs with random slope models (RS-CRSEs) had higher power than with random intercept models (RI-CRSEs) and with ordinary least square models (OLS-CRSEs) for within-cluster effect and between-cluster effect with level 1 heteroscedasticity. For the between-cluster effect with level 2 heteroscedasticity, the adjusted CR-SEs with examined models had lower power than RS-KR in VP2 conditions and higher power in VP3 conditions.

This study is consistent with previous studies that KR can largely improve the performance of REML estimates and inferences of the fixed-effect parameters in small samples, but only when the homoscedasticity assumptions are met (McNeish & Stapleton, 2016; Spilke et al., 2005). The simulation results also show that the adjusted CR-SEs can provide more accurate inferences for the between-cluster effect than the KR adjustment when heteroscedasticity exists. The findings support that using the $t$ test based on adjusted CR-SEs, an extension of the BRL correction, with the extended Satterthwaite approximated degrees of freedom can control the type I error rate close to the nominal level (Imbens & Kolesár, 2016; Pustejovsky & Tipton, 2018). When comparing the adjusted CR-SEs with the KR corrected SE, our finding is consistent with Huang and Li (2021) that under homoscedasticity conditions, OLS, RI and RS had comparable rates of power. However, under heteroscedasticity conditions, the RS-KR and RS-CRSEs had higher power than RI-CRSEs and OLS-CRSEs.

One limitation of the adjusted CR-SEs is that they are only robust to violation of the homoscedasticity assumption. Robustness is a broad notion of statistical methods guarding against all kinds of violation of distributional assumptions, such as nonnormality, heteroscedasticity and outliers (Wilcox, 2017). Although the adjusted CR-SEs, a heteroscedastic-robust estimator, performed well in the current study, they may not guard against other types of assumption violations. For example, a slight departure from normality and a few outliers can seriously influence the performance of CR-SEs (MacKinnon, 2012).

Another method to improve small-sample inferences is the bootstrap with asymptotic refinement (Colin & Miller, 2015). For example, the wild bootstrap can obtain more accurate *p* values (Colin & Miller, 2015), and the percentile bootstrap can get more accurate confidence intervals (MacKinnon, 2012). For clustered data, Cameron, Gelbach, and Miller (2008) proposed a cluster generalization of the wild bootstrap method, which has been shown to perform well in the small number of clusters. Lai (2021) compared five bootstrap confidence intervals for multilevel effect size and found that residual bootstrap with basic confidence intervals performed the best for small samples. Although these bootstrap methods can estimate the heteroscedastic-robust covariance matrices of fixed-effect parameters, it is more computationally expensive than CR-SEs with small-sample adjustments in linear regression models. Instead, it is better to use the heteroscedastic-robust test statistic in conjunction with the wild bootstrap for making accurate inferences (MacKinnon, 2012).

This study has several limitations. First, we only focused on fixed effects, so little is known about the inferences of random effects corrected by the adjusted CR-SEs. Second, only the single-parameter test was examined, and future research can compare multiple-parameter $F$ tests with KR and with CR-SEs. For example, Kowalchuk, Keselman, Algina, and Wolfinger (2004) applied the KR corrections on mixed-model $F$ tests, and they found KR can effectively control the type I error rates in small samples. Pustejovsky and Tipton (2018) extended the BRL estimator to commonly used models and multiple parameters hypothesis tests. Specifically, they used Hotelling’s $T^2$ distribution with estimated degrees of freedom ($\text {df}_\text {ABRL}$), a generalization of saddle point approximations proposed by Bell and Mccaffrey (2002). The adjusted CR-SEs was found to control the type I error rate well across conditions. However, it is still unclear how the adjusted CR-SEs will perform in the multiple-parameter hypothesis tests in this simulated context.

Future research is needed to decide if the recommendations based on the current study would be generalizable to other scenarios, such as models with many more predictors, and models with many possible random slopes. For scenarios with larger sample size, it may be computationally intensive to use KR, so we would suggest using the adjusted CR-SEs with the correctly specified model. For longitudinal models that account for autoregression, it is current unknown how these two small-sample corrections for standard error estimation would perform, so future simulation studies are needed to investigate this issue.

In applied research, there are many factors to consider when choosing among methods. For example, with models that have many level 1 predictors and possible random slopes, researchers need to use substantive knowledge to make decisions about which slopes to be fixed or random and then decide whether to use the adjusted CR-SEs or KR to correct for standard errors. The choice between random and fixed effects depends on the purpose of the statistical inference (interest in differences between the clusters vs. within-cluster effects) and the tenability of the model assumptions made for the random coefficients (Hox et al., 2018; Snijders & Berkhof, 2008). In scenarios in which researchers have theoretical reasons to believe the homoscedasticity assumption is tenable, we suggest using KR as it provides nearly unbiased standard error estimates. However, if heteroscedasticity potentially exists, then the adjusted CR-SEs should be used to guard against inflated type I error rates. Note that CR-SEs is only robust to heteroscedasticity, so it is recommended to consider using bootstrap methods together with CR-SEs for more accurate statistical inferences guarding against other model misspecifications, such as nonnormality and outliers. All these potential factors should be put into the decision of which method to use.

In conclusion, with the existence of heteroscedasticity, researchers could consider using OLS-CRSEs to account for the clustered structure if they are interested in within-cluster effects. Our simulation results suggest OLS-CRSEs could provide SE estimates with tolerable bias and control the type I error rates to the nominal level. However, for those interested in between-cluster effects and the variability of random coefficients across clusters, we recommend RS-CRSEs, which not only provides higher statistical power but also effectively controls type I error rates. The common practice for small samples, KR-RS, performs well under homoscedasticity but may yield inaccurate type I error rates for the between-cluster effects when heteroscedasticity exists.

## Open Practices Statement

Simulation codes are openly available on the project’s Open Science Framework page (https://osf.io/sa5wh/).

## Supplementary Information

Below is the link to the electronic supplementary material.

Supplementary file 1 (pdf 337 KB)

## Footnotes

1 We did not include RI and KR because while CR-SEs are designed to be robust for misspecification in the random effect structure, KR is not, and we expected that omitting random slopes would lead to underestimated standard errors with KR for the within-cluster fixed-effect coefficients. To verify our expectation, we ran a small-scale simulation and found RI and KR performed highly similar to RS and KR when estimating between-cluster effect, as both produced more problematic standard errors than the adjusted CR-SEs when level 2 heteroscedasticity existed. For within-cluster effect, RI and KR yielded smaller standard error estimates and thus larger relative bias than the other four methods. Descriptions of the simulation and the results could be found in Supplemental Materials.

2 The full data set with complete observations used by Snijders and Bosker (2011) can be accessed online from https://www.stats.ox.ac.uk/~snijders/mlbook.htm#data. The code for randomly sampling 15 schools are provided in the OSF repository (https://osf.io/sa5wh/).
