# Supplementary material for: Evaluating two small-sample corrections for fixed-effects standard errors and inferences in multilevel models with heteroscedastic, unbalanced, clustered data
Source: Behav Res Methods. 2024 Feb 6;56(6):5930–46. doi: 10.3758/s13428-023-02325-9 (PMC11541410; doi:10.3758/s13428-023-02325-9)
Supplement: Supplementary file 1 — (pdf 337 KB) [file 13428_2023_2325_MOESM1_ESM.pdf]

Appendix A

Supplemental Material

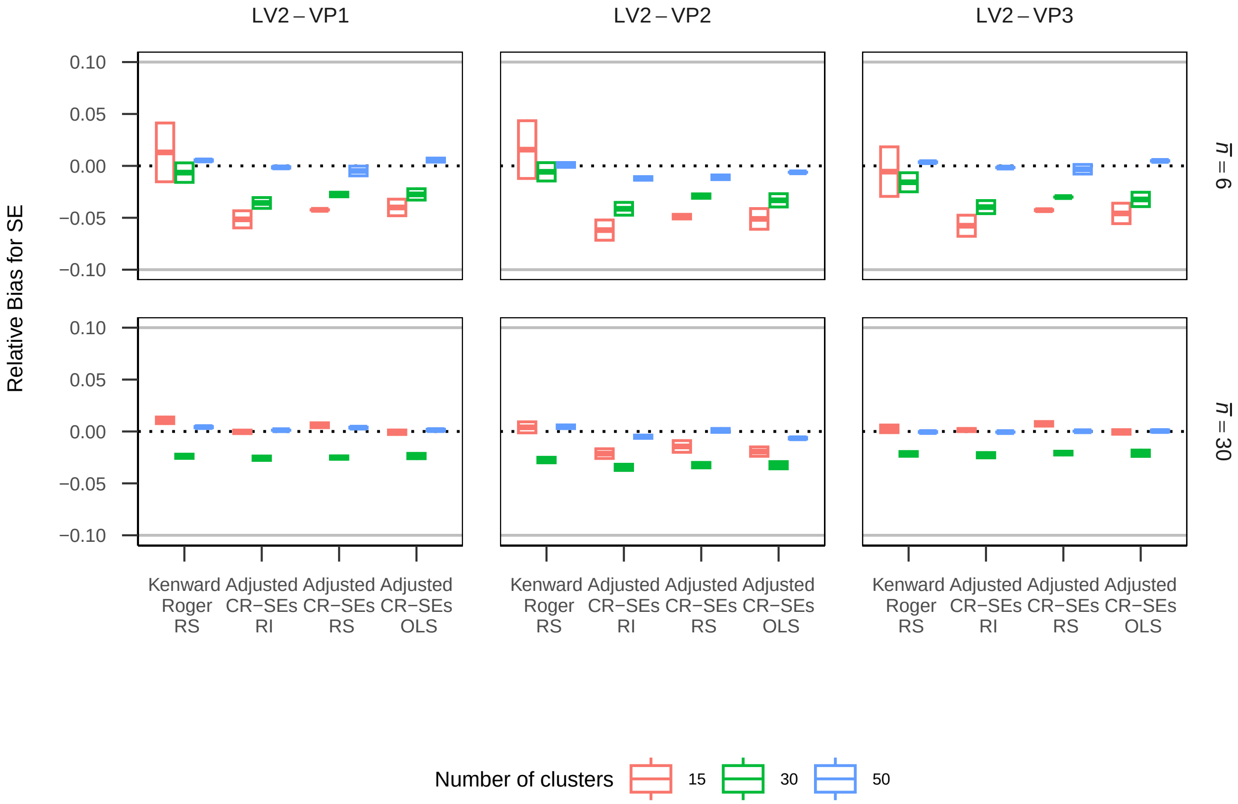

Figure S1

Relative Bias for Standard Errors of Within-Cluster Coefficient (γ_{10}) With Heteroscedasticity at Level-2. Note $\bar{n}$ is the average cluster size, SE means the standard error of the between-cluster coefficient. The gray lines represent the lower and upper bounds of acceptable values of relative bias for standard errors. VP1 represents homoscedasticity; VP2 represents when the conditional variance of outcome variable is largest when the predictors are at the average values; VP3 represents when the conditional variance of outcome variable is smallest when the predictors are at the average values. Adjusted CR-SEs is the abbreviation of the adjusted cluster-robust standard errors. OLS represents the Ordinary Least Squares models; RI represents the random intercept models; RS represents the random slope models.

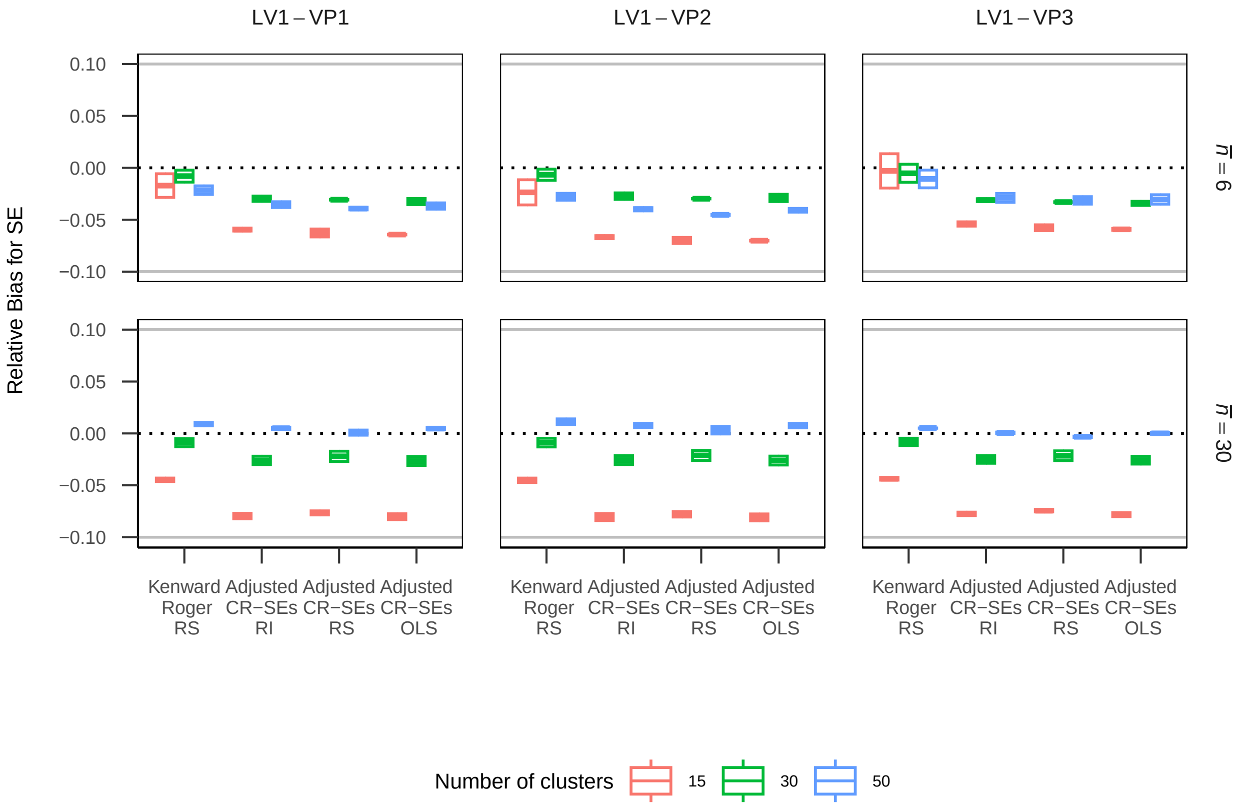

Figure S2

Relative Bias for Standard Errors of Between-Cluster Coefficient (γ_{01}) With Heteroscedasticity at Level-1. Note $\bar{n}$ is the average cluster size, SE means the standard error of the between-cluster coefficient. The gray lines represent the lower and upper bounds of acceptable values of relative bias for standard errors. VP1 represents homoscedasticity; VP2 represents when the conditional variance of outcome variable is largest when the predictors are at the average values; VP3 represents when the conditional variance of outcome variable is smallest when the predictors are at the average values. Adjusted CR-SEs is the abbreviation of the adjusted cluster-robust standard errors. OLS represents the Ordinary Least Squares models; RI represents the random intercept models; RS represents the random slope models.

Table S1

Type I Error Rates and Simulation-based Power for Detecting Between-Cluster Effect With Heteroscedasticity at Level-2

			Level-2 VP1			Level-2 VP2			Level-2 VP3		
ICC	J	Estimator	Type I	Power	Power’	Type I	Power	Power’	Type I	Power	Power’
0.1	15	Kenward-Roger RS	0.044	0.552	0.548	0.100	0.527	0.409	0.021	0.512	0.512
0.1	15	Adjusted CR-SEs RI	0.043	0.465	0.465	0.055	0.371	0.353	0.031	0.515	0.515
0.1	15	Adjusted CR-SEs RS	0.044	0.474	0.474	0.056	0.381	0.358	0.032	0.526	0.526
0.1	15	Adjusted CR-SEs OLS	0.045	0.452	0.452	0.056	0.357	0.337	0.032	0.488	0.488
0.1	30	Kenward-Roger RS	0.047	0.854	0.854	0.106	0.810	0.717	0.019	0.832	0.832
0.1	30	Adjusted CR-SEs RI	0.046	0.816	0.816	0.055	0.676	0.661	0.038	0.851	0.851
0.1	30	Adjusted CR-SEs RS	0.047	0.824	0.822	0.055	0.686	0.668	0.040	0.856	0.856
0.1	30	Adjusted CR-SEs OLS	0.046	0.803	0.802	0.055	0.645	0.627	0.038	0.830	0.830
0.1	50	Kenward-Roger RS	0.049	0.960	0.960	0.112	0.929	0.878	0.017	0.953	0.953
0.1	50	Adjusted CR-SEs RI	0.049	0.949	0.947	0.056	0.854	0.839	0.042	0.963	0.963
0.1	50	Adjusted CR-SEs RS	0.050	0.953	0.951	0.058	0.864	0.846	0.044	0.967	0.967
0.1	50	Adjusted CR-SEs OLS	0.048	0.943	0.942	0.058	0.825	0.804	0.042	0.955	0.955

Note. Type I represents Type I error rates and Power’ represents corrected power. J is the number of clusters; ICC represents the intraclass coefficient. Adjusted CR-SEs is the abbreviation of the adjusted cluster-robust standard errors. OLS represents the Ordinary Least Squares models; RI represents the random intercept models; RS represents the random slope models. VP1 represents homoscedasticity; VP2 represents when the conditional variance of outcome variable is largest when the predictors are at the average values; VP3 represents when the conditional variance of outcome variable is smallest when the predictors are at the average values.

Table S2

Type I Error Rates and Simulation-based Power for Detecting Within-Cluster Effect With Heteroscedasticity at Level-2

			Level-2 VP1			Level-2 VP2			Level-2 VP3		
ICC	J	Estimator	Type I	Power	Power’	Type I	Power	Power’	Type I	Power	Power’
0.1	15	Kenward-Roger RS	0.034	0.529	0.529	0.034	0.520	0.520	0.038	0.492	0.492
0.1	15	Adjusted CR-SEs RI	0.049	0.537	0.530	0.049	0.528	0.521	0.050	0.471	0.462
0.1	15	Adjusted CR-SEs RS	0.047	0.567	0.564	0.048	0.565	0.560	0.048	0.513	0.509
0.1	15	Adjusted CR-SEs OLS	0.049	0.532	0.528	0.048	0.523	0.518	0.049	0.465	0.461
0.1	30	Kenward-Roger RS	0.046	0.839	0.838	0.049	0.821	0.817	0.049	0.799	0.795
0.1	30	Adjusted CR-SEs RI	0.055	0.829	0.818	0.056	0.795	0.783	0.054	0.757	0.743
0.1	30	Adjusted CR-SEs RS	0.055	0.854	0.843	0.056	0.834	0.822	0.054	0.807	0.798
0.1	30	Adjusted CR-SEs OLS	0.052	0.824	0.817	0.054	0.791	0.782	0.053	0.752	0.740
0.1	50	Kenward-Roger RS	0.045	0.953	0.953	0.048	0.942	0.941	0.045	0.931	0.931
0.1	50	Adjusted CR-SEs RI	0.050	0.940	0.938	0.053	0.920	0.916	0.048	0.900	0.899
0.1	50	Adjusted CR-SEs RS	0.050	0.957	0.955	0.053	0.945	0.940	0.048	0.933	0.932
0.1	50	Adjusted CR-SEs OLS	0.050	0.938	0.937	0.052	0.917	0.915	0.049	0.895	0.894

Note. Type I represents Type I error rates and Power’ represents corrected power. J is the number of clusters; ICC represents the intraclass coefficient. Adjusted CR-SEs is the abbreviation of the adjusted cluster-robust standard errors. OLS represents the Ordinary Least Squares models; RI represents the random intercept models; RS represents the random slope models. VP1 represents homoscedasticity; VP2 represents when the conditional variance of outcome variable is largest when the predictors are at the average values; VP3 represents when the conditional variance of outcome variable is smallest when the predictors are at the average values.

Table S3

Type I Error Rates and Simulation-based Power for Detecting Between-Cluster Effect With Heteroscedasticity at Level-1

			Level-1 VP1			Level-1 VP2			Level-1 VP3		
ICC	J	Estimator	Type I	Power	Power'	Type I	Power	Power'	Type I	Power	Power'
0.3	15	Kenward-Roger RS	0.050	0.284	0.278	0.051	0.285	0.281	0.048	0.268	0.266
0.3	15	Adjusted CR-SEs RI	0.042	0.216	0.216	0.040	0.214	0.214	0.042	0.211	0.211
0.3	15	Adjusted CR-SEs RS	0.045	0.225	0.223	0.042	0.227	0.227	0.044	0.216	0.216
0.3	15	Adjusted CR-SEs OLS	0.044	0.211	0.211	0.042	0.207	0.207	0.045	0.204	0.204
0.3	30	Kenward-Roger RS	0.047	0.547	0.547	0.048	0.550	0.550	0.048	0.526	0.526
0.3	30	Adjusted CR-SEs RI	0.044	0.482	0.482	0.046	0.478	0.478	0.045	0.464	0.464
0.3	30	Adjusted CR-SEs RS	0.044	0.494	0.494	0.046	0.497	0.497	0.045	0.476	0.476
0.3	30	Adjusted CR-SEs OLS	0.044	0.462	0.462	0.047	0.452	0.452	0.045	0.442	0.442
0.3	50	Kenward-Roger RS	0.050	0.770	0.766	0.051	0.770	0.767	0.049	0.744	0.742
0.3	50	Adjusted CR-SEs RI	0.051	0.720	0.712	0.049	0.718	0.711	0.049	0.703	0.700
0.3	50	Adjusted CR-SEs RS	0.051	0.742	0.738	0.052	0.748	0.743	0.050	0.718	0.714
0.3	50	Adjusted CR-SEs OLS	0.051	0.694	0.686	0.050	0.681	0.673	0.050	0.669	0.662

Note. Type I represents Type I error rates and Power' represents corrected power. J is the number of clusters; ICC represents the intraclass coefficient. Adjusted CR-SEs is the abbreviation of the adjusted cluster-robust standard errors. OLS represents the Ordinary Least Squares models; RI represents the random intercept models; RS represents the random slope models. VP1 represents homoscedasticity; VP2 represents when the conditional variance of outcome variable is largest when the predictors are at the average values; VP3 represents when the conditional variance of outcome variable is smallest when the predictors are at the average values.

Supplementary Material B

Supplemental Simulation 1: $\tau_0^2 > \tau_1^2$

To make simulation design more closely align to applied research, we incorporated the conditions with $\tau_0^2 > \tau_1^2$ in this small-scale simulation. The same two-level random slope model with one predictor at level-1 (x_{ij}) and one predictor at level-2 (z_j) was used, and the parameter values were the same as described in the main text. Six conditions were varied in this simulation: (a) number of clusters; (b) $\gamma_{10} = \{0, 0.3\}$; (c) $\gamma_{01} = \{0, 0.3\}$; (d) variance patterns (VP) at level-1; (e) VP at level-2; and (f) relationship between τ_0^2 and τ_1^2 (equal, unequal). There were $3 \times 2 \times 2 \times 3 \times 3 \times 2 = 216$ conditions. We simulated 1,000 data sets and analyzed the simulated data using the four options mentioned in the main text (OLS-CRSEs, RI-CRSEs, RS-CRSEs, and RS-KR).

The results showed conditions with $\tau_0^2 = \tau_1^2$ and $\tau_0^2 > \tau_1^2$ perform very similarly in terms of relative bias of standard errors, Type I error rates, and power. See Figure B1 for relative bias, Figure B2 for Type I error rates, and Table B1 for power of the between-cluster coefficients (γ_{01}). The within-cluster coefficients showed the same pattern. Thus, we confirmed that it is reasonable to set $\tau_0^2 = \tau_1^2$ in our simulation design.

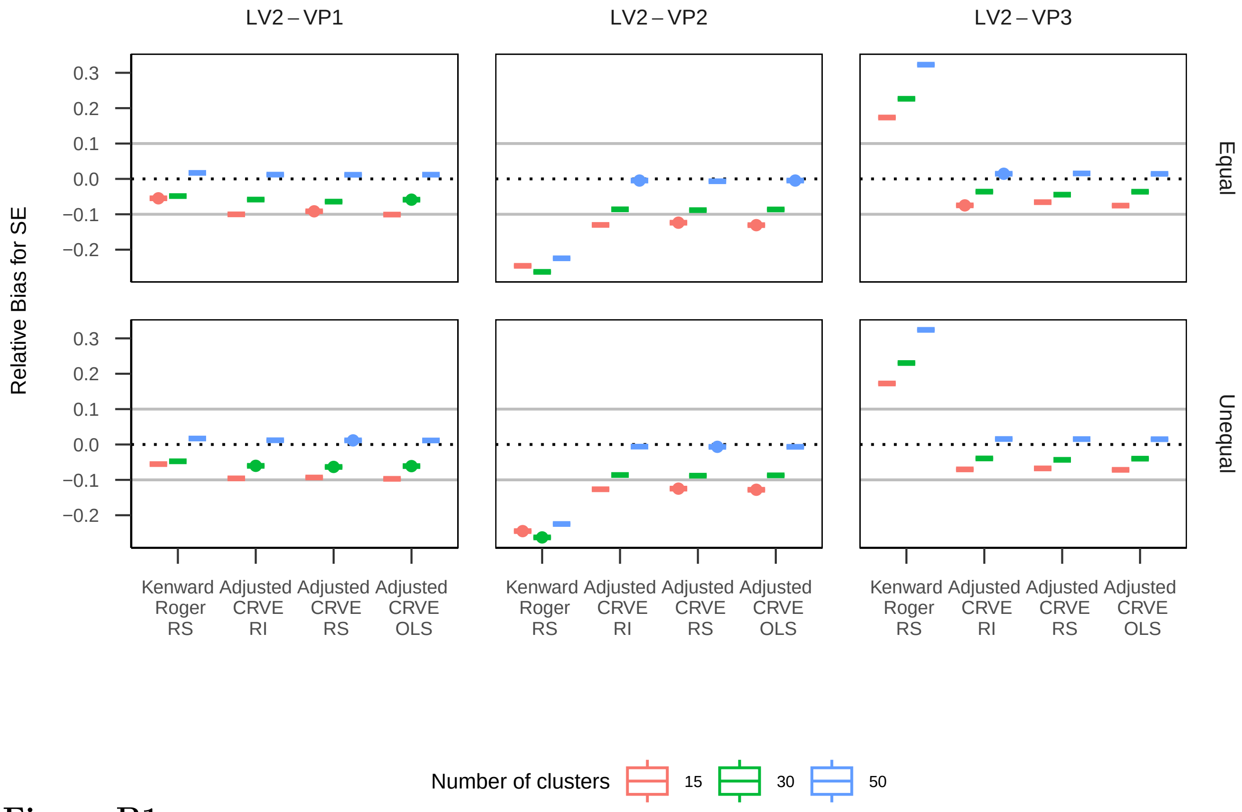

Figure B1

Relative Bias for Standard Errors of Between-Cluster Coefficient (γ_{01}) With Heteroscedasticity at Level-2 from Supplemental Simulation 1. Note "Equal" and "Unequal" correspond to conditions with $\tau_0^2 = \tau_1^2$ and $\tau_0^2 > \tau_1^2$, SE means the standard error of the between-cluster coefficient. The gray lines represent the lower and upper bounds of acceptable values of relative bias for standard errors. Adjusted CR-SEs is the abbreviation of the adjusted cluster-robust standard errors. OLS represents the Ordinary Least Squares models; RI represents the random intercept models; RS represents the random slope models. VP1 represents homoscedasticity; VP2 represents when the conditional variance of outcome variable is largest when the predictors are at the average values; VP3 represents when the conditional variance of outcome variable is smallest when the predictors are at the average values.

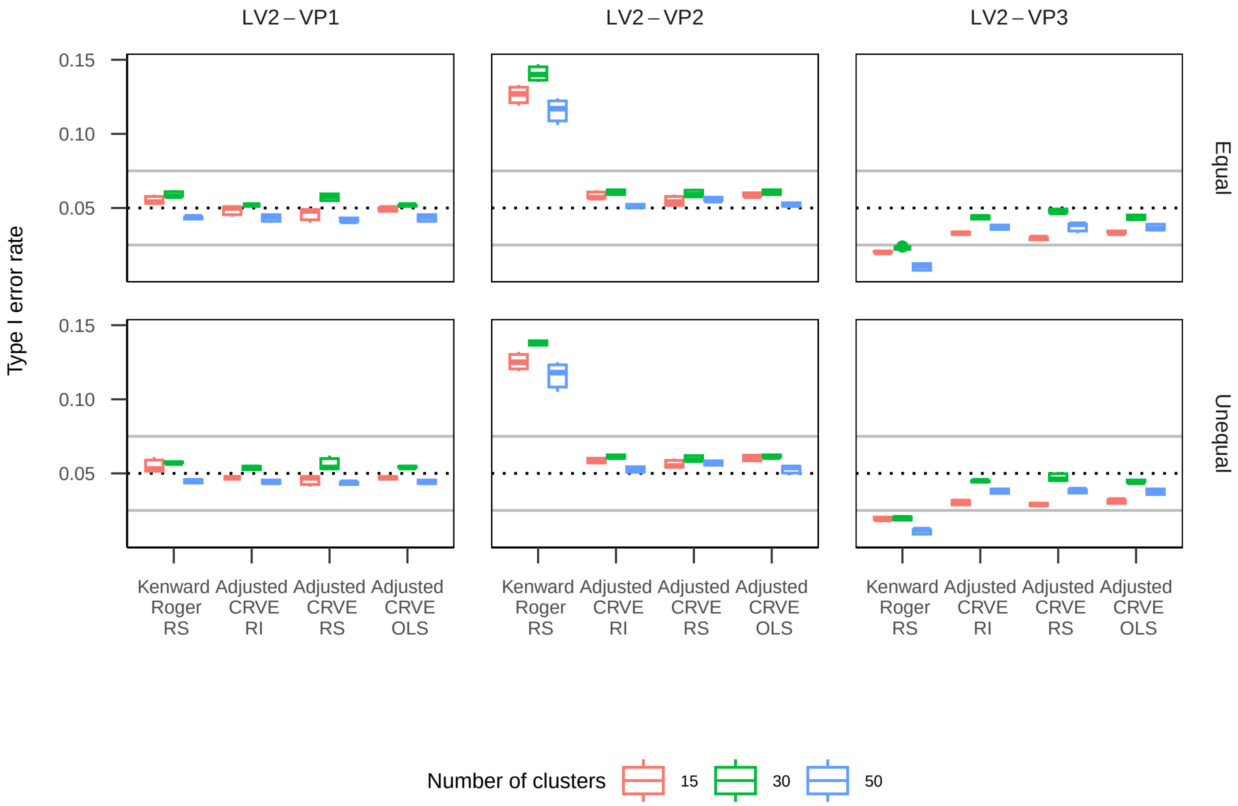

Figure B2

Type I error rates for Standard Errors of Within-Cluster Coefficient (γ_{01}) With Heteroscedasticity at Level-2 from Supplemental Simulation 1. Note "Equal" and "Unequal" correspond to conditions with $\tau_0^2 = \tau_1^2$ and $\tau_0^2 > \tau_1^2$, SE means the standard error of the between-cluster coefficient. The gray lines represent the lower and upper bounds of acceptable values of Type I error rates for standard errors. Adjusted CR-SEs is the abbreviation of the adjusted cluster-robust standard errors. OLS represents the Ordinary Least Squares models; RI represents the random intercept models; RS represents the random slope models. VP1 represents homoscedasticity; VP2 represents when the conditional variance of outcome variable is largest when the predictors are at the average values; VP3 represents when the conditional variance of outcome variable is smallest when the predictors are at the average values.

[tbp]

Table B1

Supplemental Simulation 1: Simulation-based Power for Detecting Between-Cluster Effect

		Equal		Unequal	
J	Estimator	Power	Power'	Power	Power'
15	Kenward-Roger RS	0.714	0.657	0.715	0.659
15	Adjusted CRVE RI	0.610	0.601	0.613	0.604
15	Adjusted CRVE RS	0.612	0.604	0.613	0.603
15	Adjusted CRVE OLS	0.614	0.605	0.617	0.607
30	Kenward-Roger RS	0.954	0.919	0.954	0.918
30	Adjusted CRVE RI	0.906	0.897	0.909	0.902
30	Adjusted CRVE RS	0.910	0.901	0.911	0.900
30	Adjusted CRVE OLS	0.906	0.896	0.909	0.902
50	Kenward-Roger RS	0.996	0.991	0.996	0.990
50	Adjusted CRVE RI	0.983	0.983	0.984	0.983
50	Adjusted CRVE RS	0.984	0.983	0.984	0.983
50	Adjusted CRVE OLS	0.984	0.983	0.984	0.984

Note. Power' represents corrected power; J is the number of clusters. Adjusted CR-SEs is the abbreviation of the adjusted cluster-robust standard errors. OLS represents the Ordinary Least Squares models; RI represents the random intercept models; RS represents the random slope models. Equal represents the conditions that the variance of random intercepts equal to the variance of random slopes. Unequal represents the conditions that variance of random intercepts is larger than the variance of random slopes

Supplementary Material C

Supplemental Simulation 2: Kenward-Roger With Random Intercept Model

To examine how Kenward-Roger (KR) would perform with random intercept model, we conducted the current simulation with a similar setup (simulation design, parameter values, analysis model) as the one described in main text. Five conditions were varied in the simulation: (a) number of clusters; (b) $\gamma_{10} = \{0, 0.3\}$; (c) $\gamma_{01} = \{0, 0.3\}$; (d) variance patterns (VP) at level-1; and (e) VP at level-2. We simulated 1,000 data sets for each of the 108 conditions. We fit the OLS regression using the R function `lm`, and the RI and RS models using the R package *lme4* (Bates et al., 2015). The adjusted CR-SEs was applied after fitting the OLS regression (OLS-CRSEs), the RI model (RI-CRSEs), and the RS model (RS-CRSEs) using the R package *clubSandwich* (Pustejovsky, 2021). The KR correction was applied with the RI model (RI-KR), and the RS model (RS-KR) using the R package *lmerTest* (Kuznetsova et al., 2017).

Figure C1, Figure C2 and Table C1 showed RI-KR performed similarly as RS-KR in terms of relative bias of standard errors, Type I error rates, and power for between-cluster effect (γ_{01}). For within-cluster effect (γ_{10}), the KR corrected standard errors for RI were underestimated with a larger degree than RS-KR across the level-2 variance patterns. The magnitude of relative bias in standard error estimates generated by RI-KR ranged from -0.57 to -0.43 (M = -0.51, SD = 0.04), which was a lot larger compared to the rest of the methods as shown in Table C1. Type I error rates for RI-KR were higher than the acceptable range, with magnitude ranged from 0.275 to 0.413. In contrast, RS-KR controlled Type I error rates well, with magnitude ranged from 0.038 to 0.067. RI-KR had power ranging from 0.95 to 1, whereas RS-KR had power with lower magnitude from 0.68 to 1.

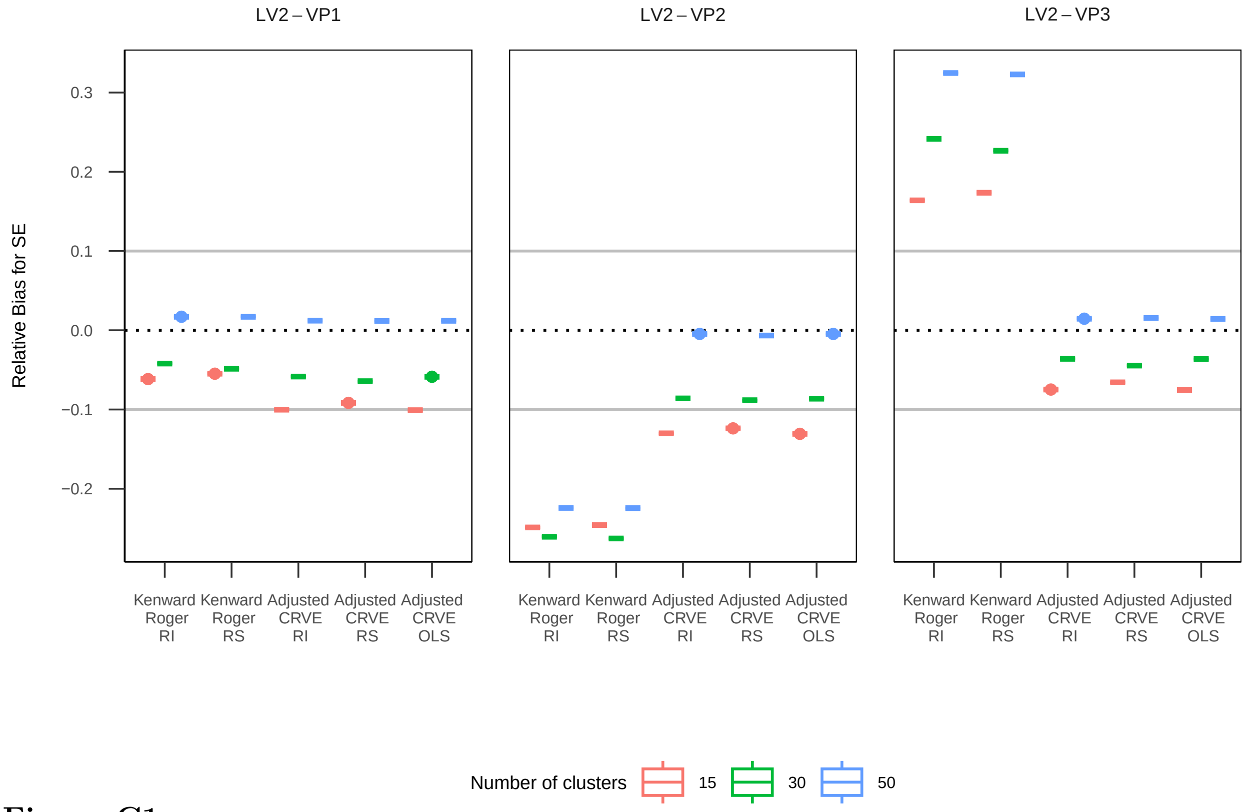

Figure C1

Relative Bias for Standard Errors of Between-Cluster Coefficient (γ_{01}) With Heteroscedasticity at Level-2 from Supplemental Simulation 2. SE means the standard error of the between-cluster coefficient. The gray lines represent the lower and upper bounds of acceptable values of relative bias for standard errors. Adjusted CR-SEs is the abbreviation of the adjusted cluster-robust standard errors. OLS represents the Ordinary Least Squares models; RI represents the random intercept models; RS represents the random slope models. VP1 represents homoscedasticity; VP2 represents when the conditional variance of outcome variable is largest when the predictors are at the average values; VP3 represents when the conditional variance of outcome variable is smallest when the predictors are at the average values.

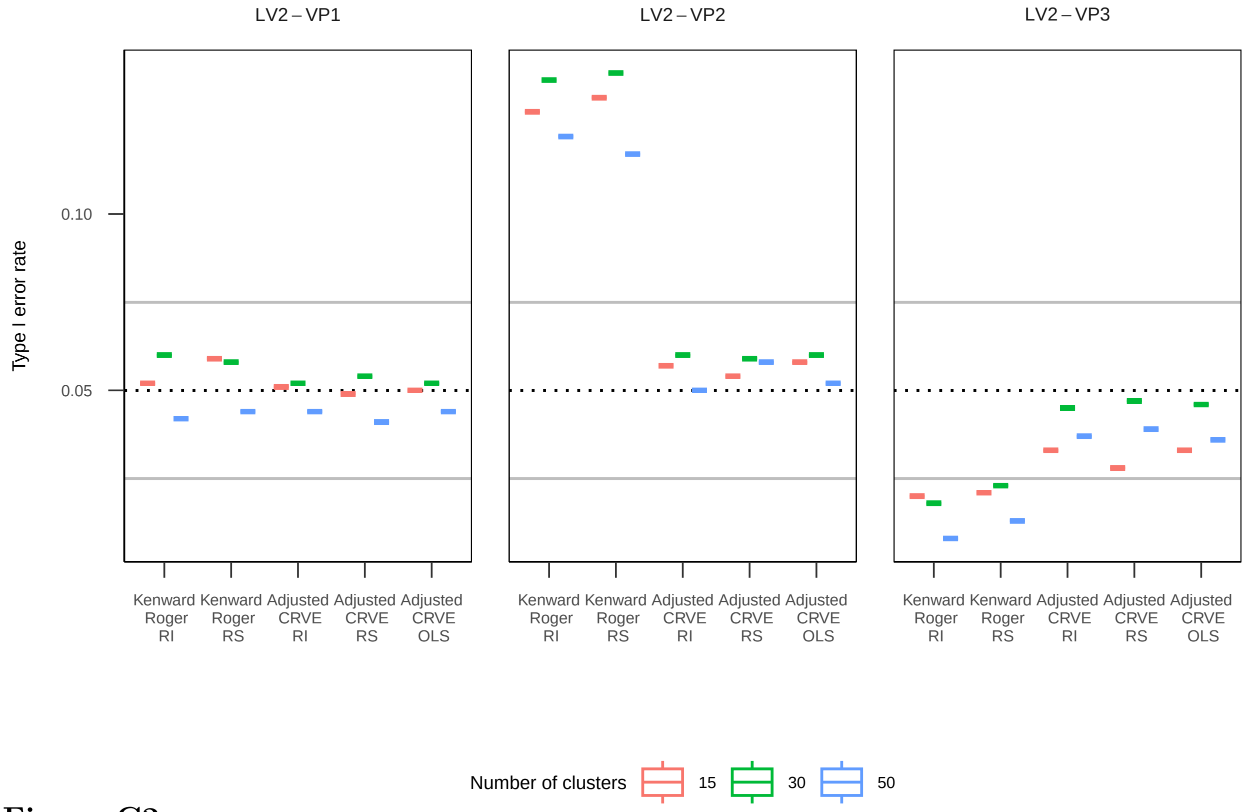

Figure C2

Type I error rates for Standard Errors of Between-Cluster Coefficient (γ_{01}) With Heteroscedasticity at Level-2 from Supplemental Simulation 2. SE means the standard error of the between-cluster coefficient. The gray lines represent the lower and upper bounds of acceptable values of Type I error rates for standard errors. Adjusted CR-SEs is the abbreviation of the adjusted cluster-robust standard errors. OLS represents the Ordinary Least Squares models; RI represents the random intercept models; RS represents the random slope models. VP1 represents homoscedasticity; VP2 represents when the conditional variance of outcome variable is largest when the predictors are at the average values; VP3 represents when the conditional variance of outcome variable is smallest when the predictors are at the average values.

Table C1

Supplemental Simulation 2: Standard Error Estimates, Relative Bias, Type I Error Rates and Power for Between-Cluster Effect

		Level-2 VP1					Level-2 VP2					Level-2 VP3				
J	Estimator	SE	Bias	Type I	Power	Power’	SE	Bias	Type I	Power	Power’	SE	Bias	Type I	Power	Power’
15	Kenward-Roger RI	0.107	-0.060	0.052	0.728	0.721	0.105	-0.246	0.124	0.703	0.546	0.114	0.162	0.018	0.706	0.706
15	Kenward-Roger RS	0.106	-0.053	0.055	0.724	0.713	0.105	-0.242	0.126	0.707	0.546	0.113	0.172	0.020	0.712	0.712
15	Adjusted CRVE RI	0.103	-0.099	0.048	0.618	0.618	0.122	-0.129	0.059	0.480	0.451	0.091	-0.074	0.033	0.733	0.733
15	Adjusted CRVE RS	0.102	-0.090	0.046	0.619	0.619	0.121	-0.122	0.055	0.487	0.462	0.090	-0.064	0.029	0.731	0.731
15	Adjusted CRVE OLS	0.103	-0.100	0.049	0.623	0.623	0.122	-0.130	0.059	0.483	0.455	0.091	-0.074	0.034	0.736	0.736
30	Kenward-Roger RI	0.074	-0.043	0.060	0.959	0.951	0.074	-0.259	0.139	0.929	0.827	0.078	0.235	0.020	0.966	0.966
30	Kenward-Roger RS	0.073	-0.050	0.059	0.961	0.954	0.073	-0.262	0.141	0.932	0.833	0.077	0.221	0.023	0.969	0.969
30	Adjusted CRVE RI	0.073	-0.059	0.052	0.936	0.935	0.091	-0.086	0.061	0.806	0.780	0.061	-0.038	0.044	0.975	0.975
30	Adjusted CRVE RS	0.072	-0.065	0.057	0.940	0.929	0.091	-0.089	0.060	0.813	0.795	0.060	-0.046	0.048	0.977	0.977
30	Adjusted CRVE OLS	0.072	-0.060	0.052	0.936	0.935	0.091	-0.087	0.061	0.807	0.778	0.061	-0.038	0.044	0.975	0.975
50	Kenward-Roger RI	0.056	0.017	0.042	0.998	0.998	0.057	-0.222	0.119	0.993	0.974	0.059	0.319	0.009	0.998	0.998
50	Kenward-Roger RS	0.056	0.017	0.043	0.998	0.998	0.056	-0.222	0.116	0.993	0.976	0.058	0.317	0.010	0.998	0.998
50	Adjusted CRVE RI	0.056	0.012	0.043	0.996	0.996	0.073	-0.004	0.051	0.955	0.954	0.045	0.014	0.037	0.999	0.999
50	Adjusted CRVE RS	0.055	0.012	0.042	0.996	0.996	0.072	-0.006	0.056	0.958	0.954	0.045	0.015	0.037	0.999	0.999
50	Adjusted CRVE OLS	0.056	0.012	0.043	0.996	0.996	0.073	-0.004	0.052	0.956	0.954	0.045	0.014	0.037	0.999	0.999

Note. SE and bias refer to standard error estimates and relative bias in standard error estimates respectively. J is the number of clusters; Type I represents Type I error rates and Power’ represents corrected power. Adjusted CR-SEs is the abbreviation of the adjusted cluster-robust standard errors. OLS represents the Ordinary Least Squares models; RI represents the random intercept models; RS represents the random slope models. VP1 represents homoscedasticity; VP2 represents when the conditional variance of outcome variable is largest when the predictors are at the average values; VP3 represents when the conditional variance of outcome variable is smallest when the predictors are at the average values.

Table C2

Supplemental Simulation 2: Standard Error Estimates, Relative Bias, Type I Error Rates and Power for Within-Cluster Effect

		Level-2 VP1					Level-2 VP2					Level-2 VP3				
J	Estimator	SE	Bias	Type I	Power	Power’	SE	Bias	Type I	Power	Power’	SE	Bias	Type I	Power	Power’
15	Kenward-Roger RI	0.054	-0.505	0.340	0.968	0.805	0.054	-0.502	0.334	0.966	0.804	0.054	-0.533	0.362	0.959	0.769
15	Kenward-Roger RS	0.103	-0.032	0.056	0.777	0.757	0.103	-0.029	0.055	0.769	0.748	0.109	-0.043	0.051	0.747	0.731
15	Adjusted CRVE RI	0.104	-0.049	0.053	0.738	0.721	0.102	-0.057	0.056	0.743	0.724	0.110	-0.053	0.049	0.710	0.709
15	Adjusted CRVE RS	0.103	-0.039	0.060	0.764	0.738	0.102	-0.048	0.060	0.761	0.728	0.109	-0.041	0.048	0.729	0.725
15	Adjusted CRVE OLS	0.104	-0.045	0.054	0.733	0.722	0.103	-0.053	0.054	0.737	0.725	0.111	-0.050	0.049	0.710	0.701
30	Kenward-Roger RI	0.037	-0.504	0.328	0.999	0.965	0.037	-0.508	0.326	0.998	0.969	0.037	-0.527	0.346	0.998	0.951
30	Kenward-Roger RS	0.072	-0.035	0.064	0.971	0.963	0.073	-0.034	0.059	0.963	0.955	0.075	-0.039	0.061	0.954	0.942
30	Adjusted CRVE RI	0.073	-0.031	0.067	0.961	0.951	0.073	-0.038	0.061	0.953	0.943	0.077	-0.034	0.062	0.944	0.933
30	Adjusted CRVE RS	0.072	-0.035	0.062	0.969	0.963	0.072	-0.040	0.059	0.961	0.952	0.076	-0.037	0.060	0.949	0.942
30	Adjusted CRVE OLS	0.074	-0.028	0.061	0.959	0.951	0.074	-0.034	0.058	0.954	0.947	0.077	-0.032	0.059	0.940	0.935
50	Kenward-Roger RI	0.029	-0.482	0.319	1.000	1.000	0.029	-0.492	0.328	1.000	0.999	0.029	-0.503	0.339	1.000	0.999
50	Kenward-Roger RS	0.056	0.035	0.040	0.999	0.999	0.057	0.034	0.041	0.998	0.998	0.058	0.029	0.040	0.998	0.998
50	Adjusted CRVE RI	0.057	0.024	0.044	0.999	0.999	0.058	0.017	0.043	0.997	0.997	0.059	0.020	0.043	0.998	0.998
50	Adjusted CRVE RS	0.056	0.035	0.042	0.999	0.999	0.057	0.032	0.042	0.998	0.998	0.058	0.029	0.039	0.998	0.998
50	Adjusted CRVE OLS	0.057	0.026	0.042	0.999	0.999	0.058	0.021	0.044	0.998	0.998	0.060	0.021	0.043	0.998	0.998

Note. SE and bias refer to standard error estimates and relative bias in standard error estimates respectively. J is the number of clusters; Type I represents Type I error rates and Power’ represents corrected power. OLS represents the Ordinary Least Squares models; RI represents the random intercept models; RS represents the random slope models. VP1 represents homoscedasticity; VP2 represents when the conditional variance of outcome variable is largest when the predictors are at the average values; VP3 represents when the conditional variance of outcome variable is smallest when the predictors are at the average values.
